# CRISPR-Sunspot: Imaging of endogenous low-abundance RNA at the single-molecule level in live cells

**DOI:** 10.7150/thno.43094

**Published:** 2020-09-02

**Authors:** Ning-He Sun, Dan-Yang Chen, Lu-Peng Ye, Gang Sheng, Jun-Jie Gong, Bao-Hui Chen, Ying-Mei Lu, Feng Han

**Affiliations:** 1 Department of Physiology, School of Basic Medical Sciences, Nanjing Medical University, Nanjing, 211166, China.; 2 Key Laboratory of Cardiovascular & Cerebrovascular Medicine, Drug Target and Drug Discovery Center, School of Pharmacy, Nanjing Medical University, Nanjing, 211166, China.; 3 Institute of Pharmacology and Toxicology, College of Pharmaceutical Sciences, Zhejiang University, Hangzhou, 310058, China.; 4 Department of Genetics, Yale University School of Medicine, New Haven, CT, USA.; 5 Department of Cell Biology, School of Medicine, Zhejiang University, Hangzhou, 310058, China.; 6 Center for Global Health of Nanjing Medical University, Nanjing, 211166, China.

**Keywords:** CRISPR/Cas9, mRNA, imaging, neuron, transcriptional activation

## Abstract

CRISPR/Cas-based mRNA imaging has been developed to labeling of high-abundance mRNAs. A lack of non-genetically encoded mRNA-tagged imaging tools has limited our ability to explore the functional distributions of endogenous low-abundance mRNAs in cells. Here, we developed a CRISPR-Sunspot method based on the SunTag signal amplification system that allows efficient imaging of low-abundance mRNAs with CRISPR/Cas9.

**Methods:** We created a stable TRE3G-dCas9-EGFP cell line and generated an Inducible dCas9-EGFP imaging system for assessment of two factors, sgRNA and dCas9, which influence imaging quality. Based on SunTag system, we established a CRISPR-Sunspot imaging system for amplifying signals from single-molecule mRNA in live cells. CRISPR-Sunspot was used to track co-localization of *Camk2a* mRNA with regulatory protein Xlr3b in neurons. CRISPR-Sunspot combined with CRISPRa was used to determine elevated mRNA molecules.

**Results:** Our results showed that manipulating the expression of fluorescent proteins and sgRNA increased the efficiency of RNA imaging in cells. CRISPR-Sunspot could target endogenous mRNAs in the cytoplasm and amplified signals from single-molecule mRNA. Furthermore, CRISPR-Sunspot was also applied to visualize mRNA distributions with its regulating proteins in neurons. CRISPR-Sunspot detected the co-localization of *Camk2a* mRNA with overexpressed Xlr3b proteins in the neuronal dendrites. Moreover, we also manipulated CRISPR-Sunspot to detect transcriptional activation of target gene such as *HBG1* in live cells.

**Conclusion:** Our findings suggest that CRISPR-Sunspot is a novel applicable imaging tool for visualizing the distributions of low-abundance mRNAs in cells. This study provides a novel strategy to unravel the molecular mechanisms of diseases caused by aberrant mRNA molecules.

## Introduction

Abnormal localization and aggregation of mRNAs are closely related to the pathological processes of a variety of diseases 1,2. Therefore, high-performance mRNA labeling and imaging can provide rich information and valuable clues for exploring biological principles, and provide a scientific basis for elucidating the mechanism of disease. Traditionally, mRNA molecules could be displayed in fixed cells using single molecule fluorescence *in situ* hybridization (smFISH) 3 with multiple fluorescent probes tiled to a single mRNA molecule. The operation process of FISH is complicated with immobilization of the sample and the probes are costly. MS2, an mRNA aptamer, has been used to track mRNA molecules in cells. However, this RNA imaging method requires destructive modification of genomic DNA or exogenous insertion of tandem MS2 sequences into mRNAs 4.

The Clustered regularly interspaced short palindromic repeats (CRISPR)-related Cas9 has been developed as a platform for genomic nucleic acid imaging 5-10. Recently, the CRISPR/Cas13 system is also emerging as one of the RNA dynamic imaging systems 11-13. By combining the Cas9 and Cas13 systems, CRISPR liveFISH can be used to simultaneously detect genomic DNA and RNA transcripts in living cells 14. Recently, a research team proposed mRNA imaging with nuclease-deactivated Cas9 (dCas9), which can recognize mRNAs under the guidance of single guide RNA (sgRNA) to image the high-abundant transcripts 15,16. However, the mRNAs encoded by most genes are present in low**-**abundance 17,18. Visualizing such mRNA molecules with high sensitivity in cells remains challenging. To achieve this purpose, it is essential to generate a more sensitive mRNA imaging method that amplifies signals from single-molecule mRNAs with a high signal-to-noise ratio.

To enable imaging of low-abundance mRNAs with a high signal-to-noise ratio, based on SunTag 19 signal amplification system, we developed a **Sun**tag-mediated **s**ingle molecule RNA sna**p**sh**ot** method which is integrated with the CRISPR/Cas9 system, named **CRISPR-Sunspot**. Our data demonstrate that CRISPR-Sunspot is a novel applicable imaging tool for visualizing the distributions of low-abundance mRNAs in live cells.

## Methods

### Generation of imaging plasmids

In the dCas9-EGFP imaging system, the plasmid CMV-dCas9-EGFP based on pcDNA3.1 (+) contained a CMV promoter, a dCas9-2 × NLS-EGFP-coding gene, an SV40 promoter and a Puro resistance gene, the sequences of which were all enclosed by two transposable arms. In particular, the widely used *Streptococcus pyogenes* dCas9 was used in the vectors of this study 20. The PiggyBac (PB) transposase-coding gene was subcloned into pcDNA3.1 (+) to construct a CMV-PBase plasmid. The plasmid TRE3G-dCas9-EGFP in the Inducible dCas9-EGFP imaging system was constructed by replacing the CMV promoter with the TRE3G inducible promoter and adding the reverse tetracycline-controlled transactivator (rtTA) coding gene. The plasmid TRE3G-dCas9-BFP in the Inducible two-color CRISPR imaging system was constructed by replacing the EGFP coding sequence with the mTagBFP gene in TRE3G-dCas9-EGFP. The two-color lentiviral plasmid PCP-EGFP-MCP-mCherry was based on a pLVX-Blast vector in which PCP-EGFP and MCP-mCherry were driven by the TRE3G promoter and separated by a T2A element.

The lentiviral plasmid of the CRISPR-Sunspot system used in the U2OS cells was constructed by replacing the SV40 promoter with the TRE3G promoter in Addgene #60910. The lentiviral scFv-sfGFP plasmid was constructed by replacing the SV40 promoter with the TRE3G promoter, inserting the rtTA gene and removing the VP64 in the Addgene #60904 plasmid. The lentiviral plasmid scFv-sfGFP-VP64 for activation was constructed by replacing the SV40 promoter with the TRE3G promoter in the Addgene #60904 plasmid, inserting a CMV-rtTA, and subcloning a sgRNA expression cassette under the U6 promoter targeting the ~60 bp position upstream of the transcription start site (TSS) of the *HBG1* gene. The TRE3G-dCas9-24 × GCN_v4 plasmid used in neurons was constructed by replacing the CMV promoter in pcDNA3.1 (+) with the TRE3G promoter and then subcloning the dCas9 and 24 × GCN_v4 coding sequences in two steps. The scFv-sfGFP plasmid was constructed by inserting the rtTA into the multiple cloning site of pcDNA3.1 (+) and then replacing the SV40 promoter with the TRE3G promoter before inserting the scFv-sfGFP coding sequence. The Xlr3b coding sequence was also subcloned into the scFv-sfGFP plasmid to construct the Xlr3b-Flag-scFv-Myc vector, in which Xlr3b-3 × Flag was separated by a P2A element from rtTA and scFv-sfGFP was replaced by scFv-Myc.

sgRNA plasmids designed to target different mRNA sequences were constructed by inserting annealed double-stranded oligonucleotides into the Bbs.I restriction sites in the pUC57-hU6-sgRNA or pUC57-mU6-sgRNA plasmid. All 3 ×, 6 ×, or 2 sets of 3 different sgRNA plasmids were constructed using Gibson assembly (New England Biolabs). The spacer sequences of negative control sgRNAs were chosen from a sequence from λ bacteriophage 16.

Finally, all sequencing confirmations of plasmids in this study were carried out using Sanger sequencing. Unless otherwise indicated, mini-preparation procedures for the plasmids in this paper were performed using an AxyPrep Plasmid Miniprep Kit (Axygen, AP-MN-P-50), and maxi-preparation procedures were performed using an EndoFree Plasmid Maxi Kit (Qiagen, 12362).

### Cell culture

U2OS (American Type Culture Collection, HTB-96) and HEK293T cells (American Type Culture Collection, CRL-11268) were maintained in DMEM (Gibco) containing 10% fetal bovine serum (Gibco) and 50 U/mL penicillin and streptomycin (Life Technologies) at 37 °C in a 5% CO_2_ incubator. To study the changes of mRNA trafficking in stress granules, cells were treated with 0.5 mM hydrogen peroxide for 1 h.

### Selection of stable cell lines

To establish stable cell lines for imaging, we transfected U2OS cells using a PB transposon system and then performed the antibiotic selection. Plasmids containing expression elements in which dCas9-EGFP was driven by the CMV or TRE3G promoter and dCas9-BFP was driven by the TRE3G promoter, were transfected into wild-type U2OS cells for selection of stable cell lines. Briefly, the dCas9-EGFP plasmids were transfected into wild-type U2OS cells. After 48 h, the cells were digested and diluted with the medium until there is only one cell per well in a 24-well plate. Then, this single cell was cultured separately in medium containing 500 ng/mL puromycin in a 24-well plate for two weeks. Finally, we obtained a series of monoclonal cell line. During this process, a confocal microscope was used to confirm whether a GFP-positive cell clone was obtained.

For the establishment of the CRISPR-Sunspot U2OS cell lines, lentivirus was used to construct a stable TRE3G-dCas9-24 × GCN_v4 cell line, which then was infected with scFv-sfGFP lentivirus. To establish cells with HBG1 activation, we transfected TRE3G-dCas9-24 × GCN_v4-expressing cells with scFv-sfGFP-VP64 lentivirus and established a series of HBG1-expressing cell clones by culturing the isolated monoclonal cells. We then quantified the mRNA levels of *HBG1* by qPCR. The screening method of these lentivirus-infected cloned cell lines is the same as the above mentioned.

### Primary neuron culture

Primary hippocampal neurons were cultured from embryonic day 18 (E18) rat. In brief, embryos were separated from rat anaesthetized with isoflurane. The hippocampi were dissected in Ca2^+^- and Mg^2+^-free Hank's balanced salt solution (Gibco), digested in trypsin-EDTA (0.25%; Gibco) with 0.025% DNase I (Roche) at 37 °C for 15 min, then incubated with horse serum (Gibco) for neutralizing the digestion of the trypsin 21. The digests were collected and centrifuged (1,000 × *g* for 5 min) then subjected to repeated suction using a pipette in medium (DMEM containing 10% horse serum and 50 U/mL penicillin and streptomycin). The dissociated cells in the supernatant were counted after centrifugation and then plated on 6-well plates precoated with poly-D-lysine and pre-added with Neurobasal Plus medium containing 2% B27 (Gibco), 2 mM GlutaMAX (Gibco) and 50 U/mL penicillin and streptomycin (Gibco). The cells were maintained at 37 °C in a 5% CO_2_ incubator. The complete medium for neurons was replaced every 3 days. All animal use procedures were approved by the Committees at Zhejiang University for the Care and Use of Laboratory Animals.

### Cell transfection

All transient transfections in these experiments were performed with the liposomal reagent in accordance with the manufacturer's instructions. Cells were seeded into 24-well plates with glass pieces 12 h before transfection. When the cell confluence reached 70-80%, 500 ng of total plasmids were transfected with 1 µL of Lipofectamine 3000 (Invitrogen) and 1 µL of p3000 reagent in Opti-MEM (Gibco). For the selection of stable cell lines, puromycin (0.5 µg/mL) or blasticidin (5 µg/mL) was added to the medium 48 h after transfection. For induction of fluorescent protein expression, the cells were treated with doxycycline at different concentration gradients for 12 h before transfection.

For mRNA imaging in U2OS cells, sgRNA plasmids were transfected into the stable cells using Lipofectamine 3000 according to the instructions. The PAMmers were transfected into the cells using Lipofectamine RNAi MAX (Invitrogen) according to the instructions.

For neuronal transfection for mRNA imaging, dissociated neurons were collected from the supernatant, counted, resuspended using Neuron Nucleofector Solution (Lonza, VPG-1003), and transfected with different plasmid mixtures using the established transfection procedures. The neurons were seeded in 6-well plates containing Neurobasal Plus medium, and the medium was replaced with fresh Neurobasal medium 2 h later following transfection. Effectene Transfection Reagent (Qiagen, 301425) and HiPerFect Transfection Reagent (Qiagen, 301704) was used to transfect PAMmers into neurons.

### Lentivirus production and transduction

HEK293T cells were seeded in 15 cm dishes (Corning). The medium was replaced with 10 mL of the complete medium after the cells reached 80% confluence. Next, 20 µg of transfer plasmids carrying the target gene, 15 µg of psPAX2, 10 µg of pMD2.G, and 130 µL of LipoFiter Liposomal Transfection Reagent (Hanbio) were vortexed with 434 µL of DMEM and incubated at room temperature for 10 min. The plasmid and liposome mixtures were then gently added to the cells. After 12 h of transfection, the supernatant was replaced with fresh medium. The supernatant containing the viral particles was harvested after 48 h and 72 h, and the lentiviruses were purified by ultracentrifugation and then aliquoted before storage at -80 °C.

### Real-time qPCR

The clonal cells with HBG1 activation were lysed, and total RNA was isolated using RNAiso Plus (TaKaRa) as described previously 22. The RNA was reverse transcribed into cDNA using PrimeScript RT Reagent Kit with gDNA Eraser (TaKaRa) according to the instructions. The RNA expression levels were quantified by qPCR using TB Green Premix Ex Taq II (Tli RNase H Plus, TaKaRa) on a CFX96 Touch Real-Time PCR Detection System (Bio-Rad). The relative mRNA expression levels between groups were calculated through normalization to *GAPDH* expression according to the ΔΔCt method.

### Western blotting

Immunoblotting was carried out in total cell lysate after the determination of protein concentrations using the DC™ Protein Assay (Bio-Rad, 5000116). Briefly, the cell lysates containing equivalent amounts of protein were loaded and separated by SDS-PAGE and immunodetected with antibodies: rabbit anti-Hemoglobin γ (1:1,000, Cell Signaling Technology, 39386), mouse anti-CaMKII (1:500, Abcam, ab22609), rabbit anti-GAPDH (1:5,000, Cell Signaling Technology, 2118). After incubation for 12 h at 4 °C, membranes were incubated with the corresponding secondary antibody. Immunoreactivity was visualized by enhanced chemiluminescence (Amersham Life Science).

### Confocal microscopy image acquisition

For mRNA imaging, cells were fixed with 4% paraformaldehyde (PFA) for 20 min at room temperature (RT) after transfection for 24 h, permeabilized in 70% ethanol at 4°C for 1 h and mounted in Vectashield mounting medium (Vector Laboratories) with DAPI. Cell imaging was performed using an Olympus FV3000 confocal microscope. For immunocytochemistry in neurons, cells were fixed with 4% PFA for 20 min at RT. Permeabilization was performed in 0.1% Triton X-100 in PBS for 10 min at RT and blocked for 1 hour in PBS containing 3% bovine serum albumin (BSA). After blocking, cells were stained for primary antibodies: anti-Myc (1:1,000, Abcam, ab9132), anti-DYKDDDDK (FLAG) Tag (1:1,500, Cell Signaling Technology, 8146), anti-MAP2 (1:300, Millipore, 05-346), anti-HA tag (1:200, Abcam, ab9110) overnight at 4 ºC in blocking buffer, followed by labeling with corresponding fluorescent secondary antibodies (1:500, Invitrogen) for 2 h at RT in blocking buffer. After staining with DAPI, cells were washed and mounted in Vectashield mounting medium (Vector Laboratories).

### Genomic DNA extraction and Sanger sequencing

The genomic DNA of clonal cells was extracted using AxyPrep Multisource Genomic DNA Miniprep Kit (Axygen, AP-MN-MS-GDNA-50) according to the instructions. For identification of dCas9-positive cells, Tks Gflex DNA Polymerase (Takara) was used for PCR with dCas9-specific primers under the following conditions: 94 °C for 1 min; 30 cycles of 98 °C for 10 s, 60 °C for 15 s, and 68 °C for 2 min. Then, the PCR products were separated on 2% agarose gels (Biowest) and recovered using an AxyPrep DNA Gel Extraction Kit (Axygen, AP-GX-250) for sequence identification.

### Single molecule fluorescence *in situ* hybridization (smFISH)

smFISH probes recognizing *HBS1L*, *HBG1*, and *Camk2a* mRNAs were designed via Stellaris Probe Designer. Fluorescent probes labeled with Quasar 570 (Biosearch Technologies) were synthesized. RNA FISH was conducted according to the instructions. Briefly, cells were fixed with RNase-free 4% PFA for 10 min and washed with PBS for twice, followed by permeabilization with 70% ethanol at 4 °C for 1 h. Probes were hybridized to cells in a hybridization buffer at 37 °C for 12 h. After hybridization, cells were mounted in Vectashield mounting medium (Vector Laboratories). Confocal microscopy was conducted using a Zeiss LSM 800 confocal microscope.

### Live cell imaging

All imaging was performed on a spinning disk confocal microscope (Olympus, DU-897D-CS0) equipped with a 60 × 1.4 NA oil immersion objective lens. For primary neurons, the movement of the scFv-sfGFP labeling of *Camk2a* mRNA was captured in proximal dendrites (10-50 μm away from the cell body) at 1 s intervals in primary neurons at DIV8. During dynamic tracking, mRNA particles that change direction are defined as bidirectional motion. When the particle trajectory during tracking is less than 5μm, it is defined as a static state. Use ImageJ to generate the dynamic Kymographs of mRNA movement. For U2OS cells, cells were seeded into 35-mm glass-bottom dishes (Nest) and transfected with imaging vectors and PAMmer for 24 h. Then, cells were imaged at imaging solution. During this, cells were maintained at 37 °C in a chamber containing 5% CO_2_. After the acquisition, the analysis process with a focus on single-particle tracking was conducted using Imaris (Bitplane).

### Single-particle tracking analysis

Single-particle tracking analysis of labeling of *HBG1* mRNA was performed by spot building function modules in Imaris (Bitplane) and the parameters were as below: the spot was created with the largest diameter at 0.6 µm, the max distance was set to 1 µm, and the max gap size was set to 1. For short-term particle movement tracking analysis, 1 s per frame was performed. Scatterplots was plotted for the step displacement (δx, δy) of single-particle tracking in 120 s. Step displacement 13 was calculated by the following formula:


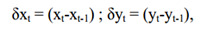


where *x_t_* and *y_t_* are the coordinate at time *t*.

For the subsequent analysis, the assigned tracks were imported into the msdanalyzer written in MATLAB for particle tracking analysis 9. The mean square displacement (MSD) as a function of time delay was measured by the following formula:


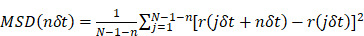


where δt is the time interval between two frames, r(t) are the coordinates at time t, N is the total number of frames and n is the number of time intervals. The diffusion coefficients D were obtained from the following formula:


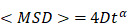


### Fluorescent signal ratio of cytoplasm/nucleus (ratio of C/N)

The specific values were calculated with the following formula and analyzed by Fiji/ImageJ.


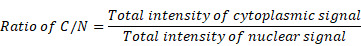


### Signal-to-noise ratio (SNR)

The signal-to-noise ratio was defined as the ratio of the fluorescence signal intensity to the background noise power. The schematic diagram of the SNR calculation was shown in Figure 4F. SNR 23 was calculated with the following formula and analyzed by Fiji/ImageJ.


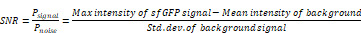


### Co-localization analysis

Co-localization analysis of *HBS1L* and *HBG1* mRNA signals labeled by CRISPR-Sunspot or smFISH assay were measured by the two separate fluorescence channels using Pearson's correlation coefficient with JACoP plugins in Fiji/ImageJ 24.

### Statistical analysis

All box plots and bar graphs were generated using the GraphPad Prism software package. All statistical analyses were conducted based on the results of biological replicates; the exact n values used to calculate statistics are indicated in the figure legends. The results are expressed as the mean ± standard error of the mean (S.E.M). The significance (*P* < 0.05) of differences between the two groups was calculated using unpaired Student's t-tests (two-tailed) or one-way ANOVA with Dunnett's multiple comparisons test.

## Results

### Optimized dCas9-EGFP system in combination with tandem sgRNA expression cassettes is a new strategy for mRNA imaging

**Figure 1:**
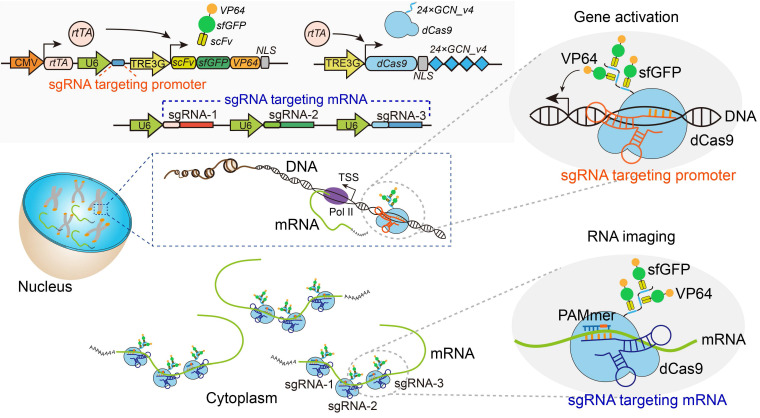
** The schematic diagram for Suntag-mediated single molecule RNA snapshot method (CRISPR-Sunspot).** For CRISPR-Sunspot, the imaging components, dCas9-24 × GCN_v4 and scFv-sfGFP proteins with NLSs were expressed under the Tet-on system for inducible control. With the guide of sgRNA and PAMmer targeting mRNA, fluorescent proteins could be recruited to single-molecule mRNA for signal amplification. In combination with CRISPR activation (CRISPRa), CRISPR-Sunspot could selectively activate transcription and illuminate the produced target mRNA molecules, simultaneously.

**Figure 2:**
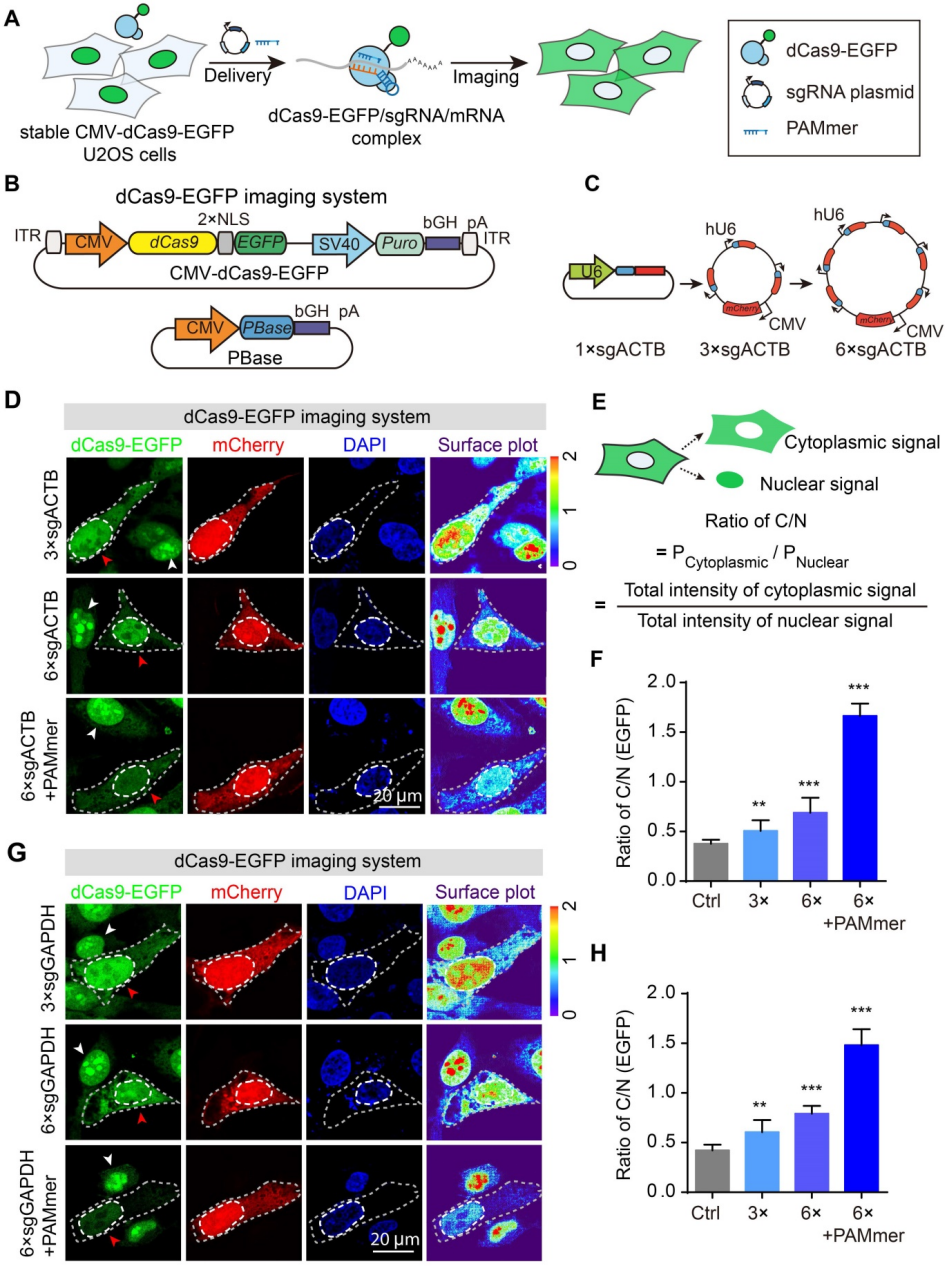
** Tandem sgRNA expression cassettes for imaging of *ACTB* or* GAPDH* mRNA in the dCas9-EGFP imaging system. (A)** Workflow for the dCas9-EGFP imaging system. Stable CMV-dCas9-EGFP U2OS cells were transfected with sgRNAs and PAMmers for subsequent mRNA imaging. **(B)** PiggyBac (PB) vector maps for constructing stable CMV-dCas9-EGFP U2OS cells. **(C)** Diagram of the expression vectors for sgRNA; 3 × or 6 × sgRNAs were co-expressed in one vector. The sgRNA targeting *ACTB* mRNA was constitutively transcribed from the human U6 polymerase III promoters, and mCherry, which was used to label the transfected cells, was under the control of the CMV promoter.** (D)** dCas9-EGFP imaging of *ACTB* mRNA in stable CMV-dCas9-EGFP U2OS cells using different sets of sgRNAs with or without PAMmers. The gray dotted lines delineate the cellular boundaries, and the white dotted lines delineate the cellular nuclei. Each inset (right panel) shows a surface plot representing a two-dimensional graph of the intensity of dCas9-EGFP. Scale bars, 20 µm.** (E)** Schematic diagram for the calculation of fluorescence ratio of cytoplasmic-to-nuclear (ratio of C/N). **(F)** Quantification of the EGFP intensity for labeling of *ACTB* mRNA based on the ratio of C/N (n = 104, 90, 96, 96 cells). **(G)** dCas9-EGFP imaging of *GAPDH* mRNA in stable CMV-dCas9-EGFP U2OS cells using different sets of sgRNAs with or without PAMmers. Scale bars, 20 µm. **(H)** Quantification of the EGFP intensity for labeling of *GAPDH* mRNA based on the ratio of C/N (n = 88, 98, 121, 110 cells). The data are displayed as the mean ± S.E.M. An unpaired t test was used. **P < 0.01, ***P < 0.001.

The imaging components for the construction of the CRISPR-Sunspot system we present here and the applications in our study are shown in Figure 1. To improve mRNA imaging efficiency, we initially investigated two factors, sgRNA and dCas9, using a basic dCas9-EGFP imaging system. We used PB transposon 25 to obtain stable CMV-dCas9-EGFP U2OS cells (Figure 2A-B and Figure S1A-B). dCas9-EGFP proteins with nuclear localization signal (NLS) was enriched in the nucleolus (including in the mitotic phase) with a partial leaked cytoplasmic signal, indicating that an excess of dCas9-EGFP proteins was expressed under the initiation of CMV (Figure S1C-D). Following post-transfection of the 1 × sgACTB (Figure 2C) with PAMmer targeting the mRNA of *ACTB*, no obvious cytoplasmic EGFP signal was observed compared to the nucleus.

Given that the U6 promoter was a weak promoter that drove fewer transcripts of sgRNA 26, we speculated that the amount of dCas9-EGFP/sgRNA complex was insufficient for cytoplasmic mRNA detection. In previous studies, tandem sgRNA cassettes in one vector was a useful strategy for co-expressing multiple sgRNAs 27,28. Therefore, the sgRNA vectors were expanded by inserting up to 3 or 6 tandem repetitions of sgRNA cassettes to increase its expression and mCherry was added to indicate the transfected cells in our study (Figure 2C). Notably, a significant reduction of nucleolar EGFP signals accompanied by the cytoplasmic localization pattern of EGFP signal was observed in mCherry^+^ cells (red arrowhead), indicating the effective and sufficient binding of dCas9 to mRNA with sgRNA and PAMmer. In contrast, the mCherry^-^ cells (white arrowhead) containing no sgRNA showed mostly nuclear retention of EGFP signal (Figure 2D). The localization of fluorescent proteins measured by the fluorescent signal ratio of cytoplasm/nucleus (C/N) (Figure 2E) showed a remarkably increasing as the sgRNA expression cassettes increases (Figure 2F). A similar pattern was also observed when sgRNA targeted *GAPDH* mRNA (Figure 2G-H). Our data demonstrate that the degree of sgRNA expression was an important factor determining the efficiency of mRNA imaging. Thus, we adopted a tandem sgRNA overexpression strategy within the CRISPR-Sunspot system.

### Inducible Tet-on system manipulating dCas9-EGFP expression improves mRNA imaging efficiency

**Figure 3:**
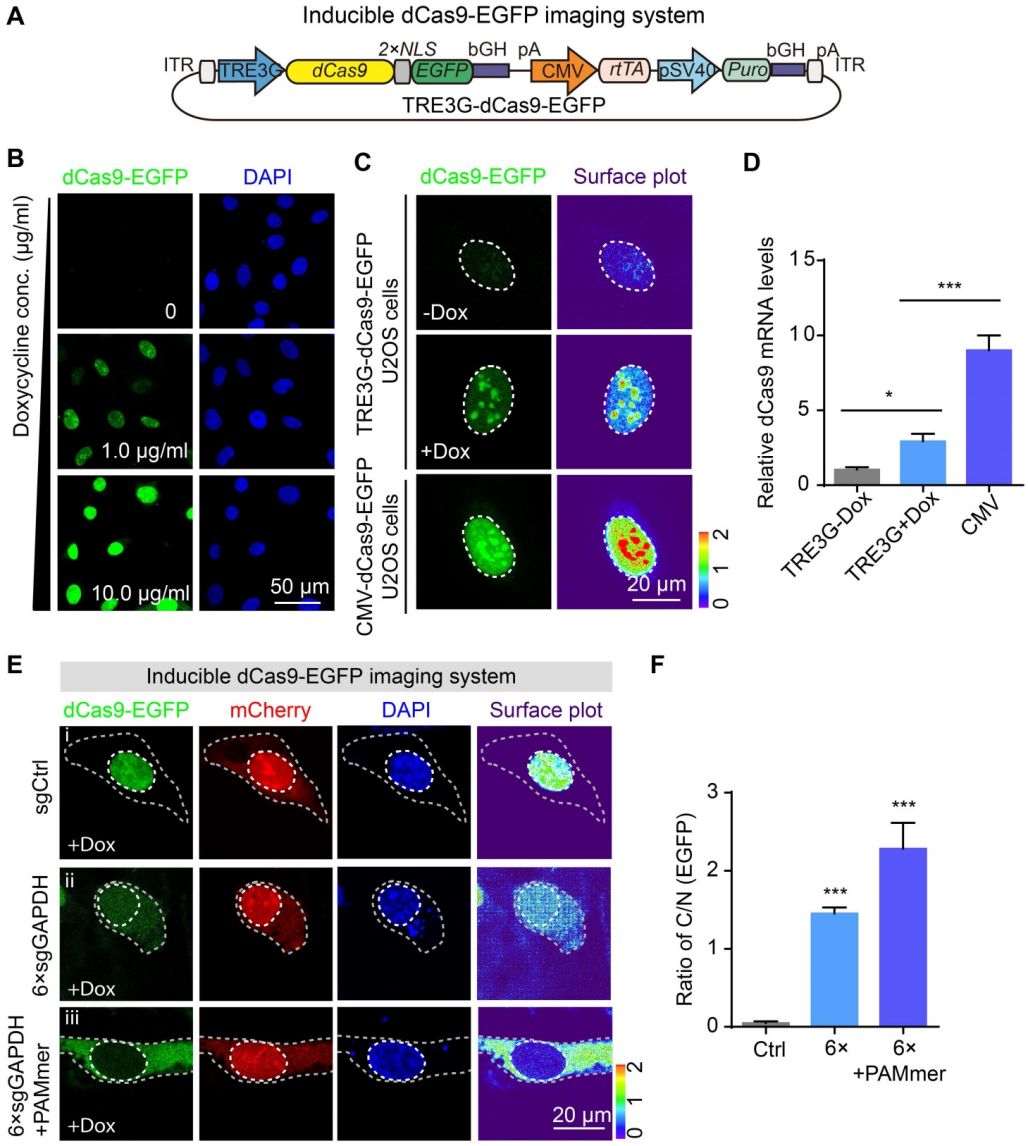
** Inducible dCas9-EGFP imaging system and tandem sgRNA expression cassettes for mRNA imaging. (A)** Diagram of the expression vectors for the Inducible dCas9-EGFP imaging system. The expression of dCas9 fused with two NLSs and EGFP was under the control of the TRE3G promoter. This same plasmid contained the rtTA expression cassette under the control of CMV promoter. **(B)** dCas9-EGFP (green) was expressed under the TRE3G promoter in the presence of different concentration of doxycycline. Scale bars, 50 µm. **(C)** Representative images of dCas9-EGFP expression driven by the CMV or TRE3G promoter. Each inset (right panel) shows a surface plot representing a two-dimensional graph of the intensity of dCas9-EGFP. Scale bars, 20 µm. **(D)** mRNA levels of dCas9-EGFP (normalized to dCas9-EGFP expression driven by the TRE3G promoter without doxycycline incubation) in U2OS cells as quantified by RT-qPCR (n = 3). **(E)** Representative images of *GAPDH* mRNA observed in stable TRE3G-dCas9-EGFP U2OS cells transfected with sgRNAs or PAMmers. dCas9-EGFP (green), mCherry reporter (red) localization, and nuclei (DAPI, blue) are visible in U2OS cells. The gray dotted lines delineate the cellular boundaries, and the white dotted lines delineate the cellular nuclei. Each inset (right panel) shows a surface plot representing a two-dimensional graph of the intensity of dCas9-EGFP. Scale bars, 20 µm. **(F)** Quantification of the EGFP intensity for labeling of *GAPDH* mRNA based on the ratio of C/N (n = 126, 102, 99 cells). The data are displayed as the mean ± S.E.M. One-way ANOVA with Dunnett's multiple comparisons test (D) and an unpaired t test (F) were used. *P < 0.05, ***P < 0.001.

To reduce the initial background signal in the cytoplasm, we controlled the expression of dCas9-fluorescent proteins by using an inducible Tet-On strategy 5, referred to as Inducible dCas9-EGFP imaging system (Figure 3A). dCas9-EGFP expression was gradually enhanced in proportion to the increased doxycycline concentration in the stable TRE3G-dCas9-EGFP cell line (Figure 3B). Indeed, dCas9-EGFP was retained in the nucleus without additional signals in the cytoplasm in TRE3G-dCas9-EGFP cells treated with 1.0 μg/mL doxycycline, which was suitable for RNA imaging compared to the CMV-dCas9-EGFP cells (Figure 3C-D). Consequently, we transfected the TRE3G-dCas9-EGFP cells with vectors containing tandem sgRNA cassettes and PAMmer to assess the imaging capability. Notably, we observed that partial EGFP proteins distributed into the cytoplasm with mRNA in the sgRNA-only group (Figure 3E, i-ii), which is consistent with the previous observations, indicating that sgRNA alone can achieve mRNA targeting, whereas PAMmer can enhance the binding of dCas9 protein to RNA 15,16. Furthermore, after cotransfection of 6 × sgRNA vectors and PAMmer, we observed that almost all dCas9-EGFP protein is present in the cytoplasm (Figure 3E, iii, and F).

Previous studies have enabled multicolor imaging of distinct DNA loci in the genome by engineering sgRNA scaffold using CRISPR 27,29,30. To verify the reliability of the inducible CRISPR mediated RNA imaging, we therefore rationally designed an Inducible two-color CRISPR imaging system for RNA imaging in which dCas9-BFP, PCP-EGFP, and MCP-mCherry were co-expressed under TRE3G promoter. In addition, sgRNA scaffolds were fused with MS2 or PP7 stem-loops (Figure S2). Interestingly, cotransfection of 3 × modified sgRNAs (Figure S3A) vectors with PAMmers resulted in a significant increase in BFP, EGFP and mCherry signal in the cytoplasm compared to control (Figure S3B, i-ii). A similar pattern with increased imaging efficiency was observed in the 6 × modified sgRNAs vectors with PAMmers groups (Figure S3B, iii, and C).

In summary, these results show that our approach manipulating the expression of fluorescent proteins tagged dCas9 increased the efficiency of RNA imaging in cells. Thus, the strategy for the inducible expression of dCas9-fluorescent proteins was also adopted in our CRISPR-Sunspot system.

### CRISPR-Sunspot system enables imaging of endogenous RNA transcripts

**Figure 4:**
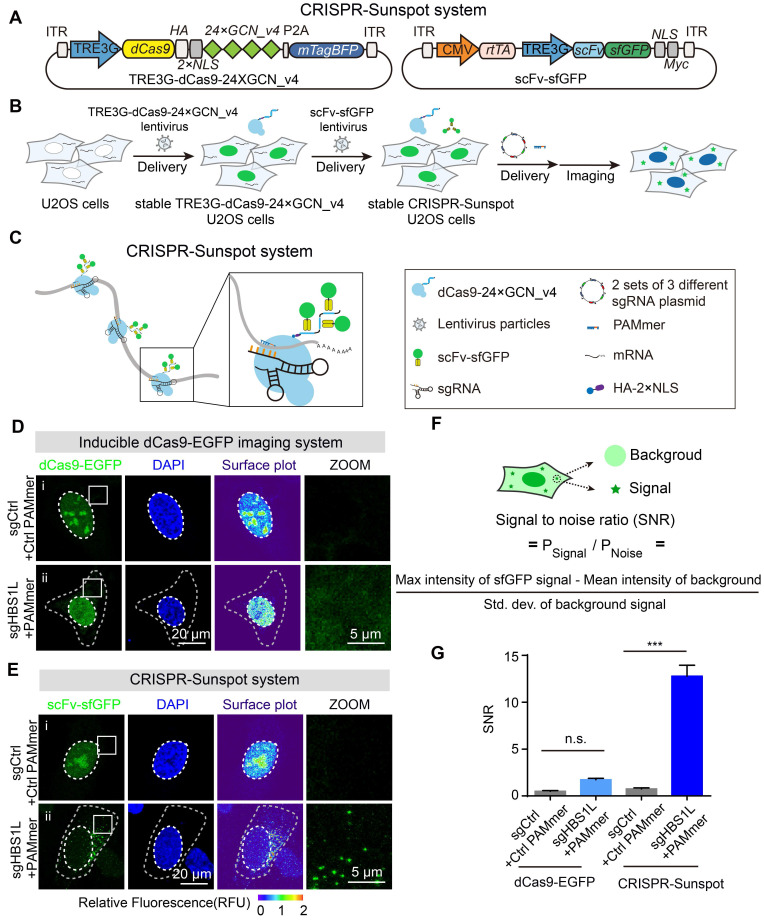
** CRISPR-Sunspot system for imaging of endogenous mRNAs. (A)** Lentiviral vector maps for CRISPR-Sunspot. The expression of dCas9 fused with an HA tag and two NLSs followed by peptide epitopes that contained 24 × GCN_v4, was under the control of TRE3G promoter. scFv fused with sfGFP was expressed under the TRE3G promoter. The reverse tetracycline-controlled transactivator (rtTA) was constitutively expressed via the CMV promoter. **(B)** Workflow for CRISPR-Sunspot. U2OS cells were infected with TRE3G-dCas9-24 × GCN_v4 lentivirus to construct stable cell lines. Stable TRE3G-dCas9-24 × GCN_v4 U2OS cells were infected with scFv-sfGFP lentivirus to construct stable CRISPR-Sunspot U2OS cells. Next, 2 sets of 3 different sgRNA plasmid and PAMmers were transfected into cells for subsequent single-molecule mRNA imaging in the cytoplasm. **(C)** Schematic diagram showing CRISPR-Sunspot imaging strategies with three target sites in one mRNA. Fluorescence signal amplification was performed with the SunTag system. **(D)** Representative images of *HBS1L* mRNA labeling using Inducible dCas9-EGFP imaging system. Scale bars, 20 µm and 5 µm (right panel). **(E)** Representative images of *HBS1L* mRNA labeling using the CRISPR-Sunspot system. CRISPR-Sunspot produced a pattern of scattered puncta. Scale bars, 20 µm and 5 µm (right panel). **(F)** Schematic diagram for the calculation of signal-to-noise ratio (SNR). **(G)** Quantification of the SNR for the two systems in D and E (n = 99, 92, 126, 92 cells). The data are displayed as the mean ± S.E.M. One-way ANOVA with Dunnett's multiple comparisons test was used. ***P < 0.001, n.s. not significant.

For CRISPR-Sunspot, we used lentivirus to obtain a stable cell line in which dCas9-24 × GCN_v4 and scFv-sfGFP proteins with NLSs were expressed under the TRE3G promoter for inducible control (Figure 4A-B). The scFv-sfGFP proteins could be recruited to the 24 × GCN_v4 tags to achieve signal amplification 19. To further amplify the signal from a single-molecule mRNA, a vector containing 2 sets of 3 different sgRNA expression cassettes for targeting 3 sites of the single mRNA molecule was designed (Figure 4C). We cotransfected sgRNA vectors and PAMmers into stable cells and then performed mRNA imaging in the cytoplasm.

To test the imaging capability of the CRISPR-Sunspot system, we chose *HBS1L* as our first target gene and sgHBS1L with PAMmers was cotransfected into cells. Notably, the nucleolus signal was attenuated but there was no obvious cytoplasmic signal while using the previous Inducible dCas9-EGFP imaging system in cells (Figure 4D). However, fluorescent spots reflecting the distributions of mRNA molecules could be detected in the cytoplasm by using the CRISPR-Sunspot system (Figure 4E). The signal-to-noise ratio (SNR) is also calculated and quantified (Figure 4F). We confirmed that the CRISPR-Sunspot system achieved a higher signal-to-noise ratio for mRNA labeling than dCas9-EGFP (Figure 4G). These results suggest that the CRISPR-Sunspot system could significantly amplify signals from mRNAs with a high SNR.

**Figure 5:**
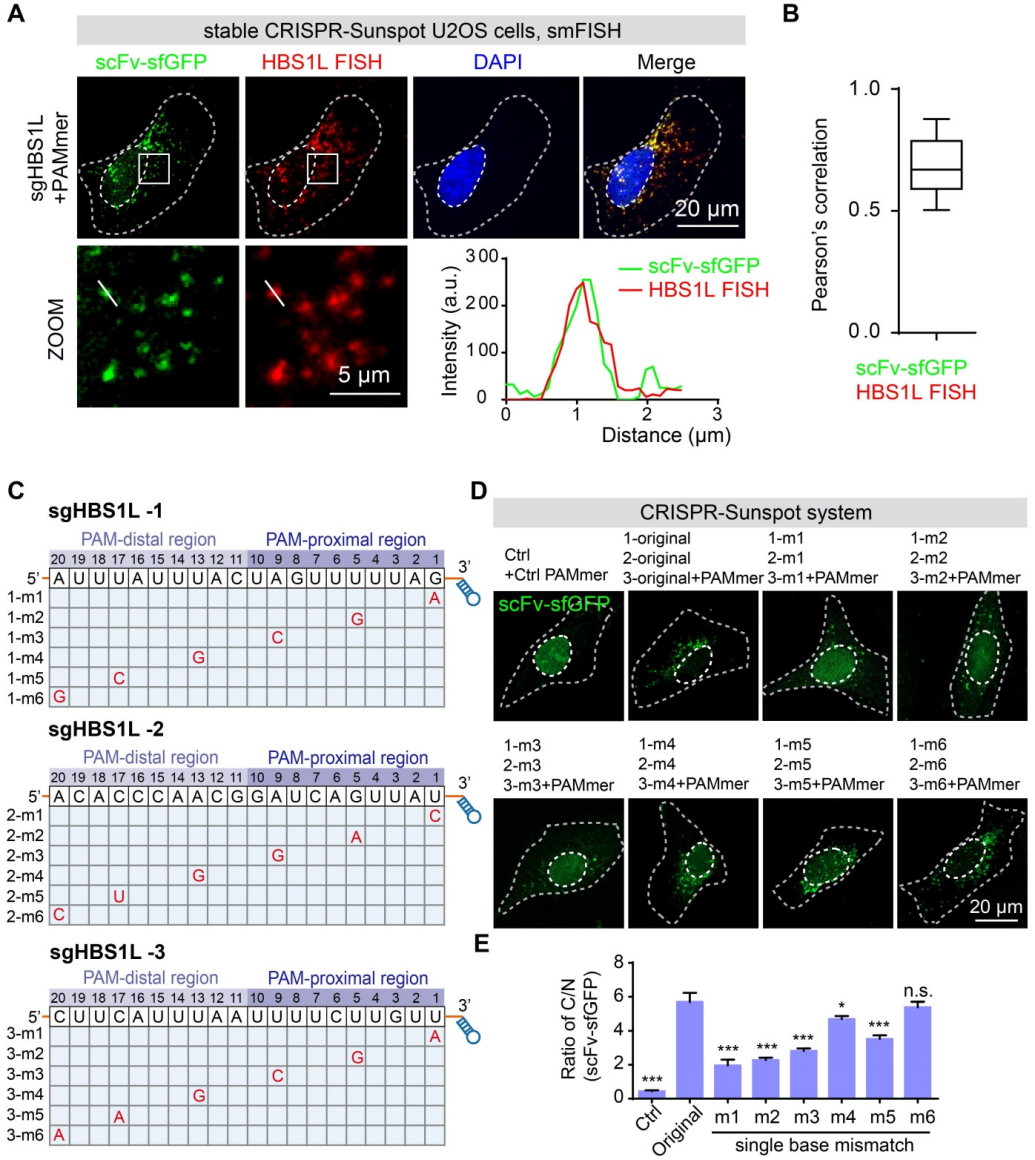
** The correlation between the signals of CRISPR-Sunspot and FISH for *HBS1L* mRNA. (A)** smFISH confirms *HBS1L* mRNA signals labeling by the CRISPR-Sunspot system in stable CRISPR-Sunspot U2OS cells. Plots of the arbitrary units (a.u.) along the line indicated the fluorescence intensity in the high-magnification images. Scale bars, 20 µm and 5 µm (lower panel).** (B)** Co-localization analysis between scFv-sfGFP targeting *HBS1L* mRNA with smFISH labeling of *HBS1L* mRNA, quantified by Pearson's correlation. n = 48 cells. **(C)** Schematic diagram of the single nucleotide mismatch in mutant sgHBS1L. **(D)** Representative images of CRISPR-Sunspot labeling of *HBS1L* mRNA with mutant sgRNAs containing single nucleotide mismatch. Original represents unmutated sgRNA. Scale bars, 20 µm. **(E)** The ratio of C/N statistics showing the effects of mutant sgHBS1L on CRISPR-Sunspot imaging efficiency. n = 48, 32, 32, 72, 74, 72, 62, 42 cells. The data are displayed as the mean ± S.E.M. An unpaired t test was used. *P < 0.05, ***P < 0.001, n.s. versus original sgRNA.

To validate the specificity of our CRISPR-Sunspot fluorescence labeling system for RNA imaging, we further performed single molecule fluorescence *in situ* hybridization (smFISH) in U2OS cells. CRISPR-Sunspot targeted *HBS1L* mRNA (scFv-sfGFP) and smFISH labeling of *HBS1L* mRNA (Quasar 570) showed co-localization (Figure 5A), which validated by Pearson's correlation (Figure 5B). Therefore, smFISH confirmed the accuracy of the CRISPR-Sunspot system for imaging the single molecule mRNA in stable CRISPR-Sunspot U2OS cells.

To estimate the off-target binding, we have also designed variant sgRNAs bearing one nucleotide mismatch in the spacer region of sgRNA bound to the mRNA to examine the RNA-binding specificity of dCas9 for RNA imaging (Figure 5C). Previous studies showed that the specificity of SpCas9 is mainly determined by the 'seed sequence', a sequence about 10 nt close to PAM (PAM-proximal region) 7,31. In our CRISPR-Sunspot imaging system, we found that single nucleotide mismatch at the PAM-distal region could be tolerated, whereas mismatches in the PAM-proximal significantly attenuated the ratio of C/N (Figure 5D-E). Therefore, we avoided off-target using established sgRNA design tools especially to minimize the possibility of off-target in the PAM-distal region when designing sgRNA target sites. Taken together, our results demonstrate the high specificity and efficiency of RNA recognition by CRISPR-Sunspot.

### CRISPR-Sunspot system achieves mRNA imaging in neurons

The transport and subcellular distributions of mRNAs are extremely critical for spatiotemporal regulation of gene expression in neurons 32. A variety of neuropsychiatric disorders are caused by the abnormal location of mRNAs in neurons. For example, trans-active response DNA-binding protein 43 (TDP-43), an RNA-binding protein, is related to amyotrophic lateral sclerosis, as it promotes transport of *Nefl* mRNA 33. Microsatellite repeat expansions encoding toxic repetitive RNA have also been found in patients with Huntington's disease 34. Therefore, high-performance mRNA imaging would have tremendous investigatory and diagnostic utility for neuropsychiatric diseases 35,36.

To further test the value of CRISPR-Sunspot in neurons, we first applied it to image primary neuronal *ACTB* mRNA granules at the single cell level (Figure S4A). dCas9-24 × GCN_v4 and scFv-sfGFP were expressed under the TRE3G promoter, and sgACTB was transcribed under the U6 promoter (Figure S4B). These vectors were cotransfected into isolated primary neurons by electrotransfection (Figure S4C). Notably, after transfection of neurons with sgRNA vectors and PAMmers targeting the *ACTB* mRNA, green fluorescence puncta were observed in dendrites in the sgACTB with PAMmers group compared to the control group (Figure S4D), suggesting that originally diffuse scFv-sfGFP proteins were recruited to dCas9/sgRNA/mRNA complexes as granules. These results suggest that CRISPR-Sunspot is suitable for mRNA imaging in neurons.

### CRISPR-Sunspot imaging system reveals the co-localization of endogenous *Camk2a* mRNA and Xlr3b protein in neurons

**Figure 6:**
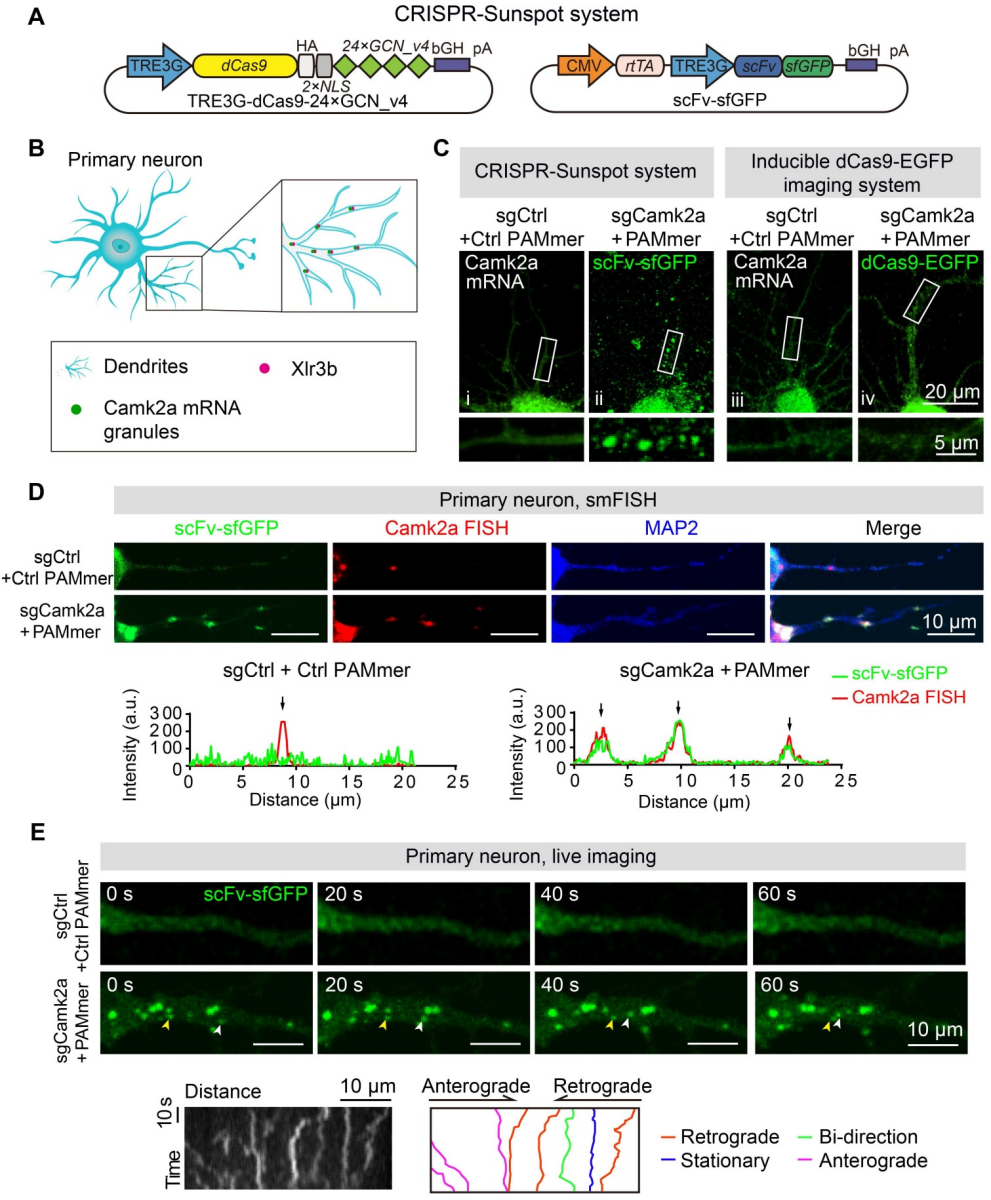
** Imaging of endogenous mRNAs in live neurons with CRISPR-Sunspot. (A)** Vector maps for CRISPR-Sunspot imaging *Camk2a* mRNA in primary neurons. dCas9-24 × GCN_v4, scFv-sfGFP, and sgCamk2a with PAMmer to examine the distribution of *Camk2a* mRNA in neurons.** (B)** Diagram of the localization of *Camk2a* mRNA and Xlr3b protein in primary neurons. **(C)** Representative images of *Camk2a* mRNA labeling using the CRISPR-Sunspot system or Inducible dCas9-EGFP imaging system in neurons. Scale bars, 20 µm and 5 µm (lower panel). Confocal images showed *Camk2a* mRNA in dendrites (indicated by sfGFP).** (D)** smFISH confirms *Camk2a* mRNA signals labeled by the CRISPR-Sunspot system in neurons. Plots of the arbitrary units (a.u.) along the dendrites indicated the fluorescence intensity in the high-magnification images. Scale bars, 10 µm. **(E)** Time-lapse images of scFv-sfGFP labeling of *Camk2a* mRNA in a dendrite of a cultured neuron at day 8 *in vitro*. Granules moved in both anterograde (yellow arrowhead) and retrograde (white arrowhead) directions. Scale bars, 10 µm. Representative kymograph indicated the movement of scFv-sfGFP labeling of *Camk2a* mRNA in a proximal dendrite. Scale bars, 10 µm (x-axis) and 10 s (y-axis). The movement of granules was shown in anterograde, retrograde, and bidirectional directions, or stationary state. Scale bars, 10 µm. See also Video S1 and S2.

Ca^2+^/calmodulin-dependent protein kinase 2a (Camk2a) is essential for learning, memory and long-term potentiation 37. Selective binding proteins govern the distributions of *Camk2a* mRNA molecules in various neuropsychiatric disorders. We used CRISPR-Sunspot with dCas9-24 × GCN_v4, scFv-sfGFP, and sgCamk2a (Figure 6A) to examine the distribution of *Camk2a* mRNA in neurons (Figure 6B). Notably, after cotransfection of neurons with sgCamk2a and PAMmers targeting endogenous *Camk2a* mRNA, scFv-sfGFP puncta representing mRNA molecules were found in dendrites with the CRISPR-Sunspot imaging system (Figure 6C, i-ii) but not with the Inducible dCas9-EGFP imaging system (Figure 6C, iii-iv). Our results indicate that CRISPR-Sunspot is capable of amplification of *Camk2a* mRNA signals in neurons. Furthermore, our smFISH results showed that the distribution patterns of scFv-sfGFP targeting *Camk2a* mRNA in neurons also correlates well with smFISH labeling of *Camk2a* mRNA (Figure 6D). To exclude the possibility that CRISPR-Sunspot might alter the target mRNA abundance or amount of translated protein, we then assessed the expression level of target genes. Furthermore, we observed no significant differences in mRNA levels among CRISPR-Sunspot targeting *Camk2a* mRNA in neurons compared to the sgControl groups (Figure S4E), implicating that CRISPR-Sunspot did not alter the target mRNA abundance. Consistently, western blot analysis showed that CRISPR-Sunspot also did not alter the amount of translated protein (Figure S4F).

To test whether our imaging methods also provided opportunities to visualize mRNA targets in live neurons, next, we performed time-lapse imaging with CRISPR-Sunspot labeling of mRNA in neurons (Figure 6E). We investigated the movement of the *Camk2a* mRNA (scFv-sfGFP labeling) in proximal dendrites (10-50 μm away from the cell body) in primary neurons and found that mRNA granules moved in anterograde and retrograde directions (Figure 6E, also see Video S1 and S2).

**Figure 7:**
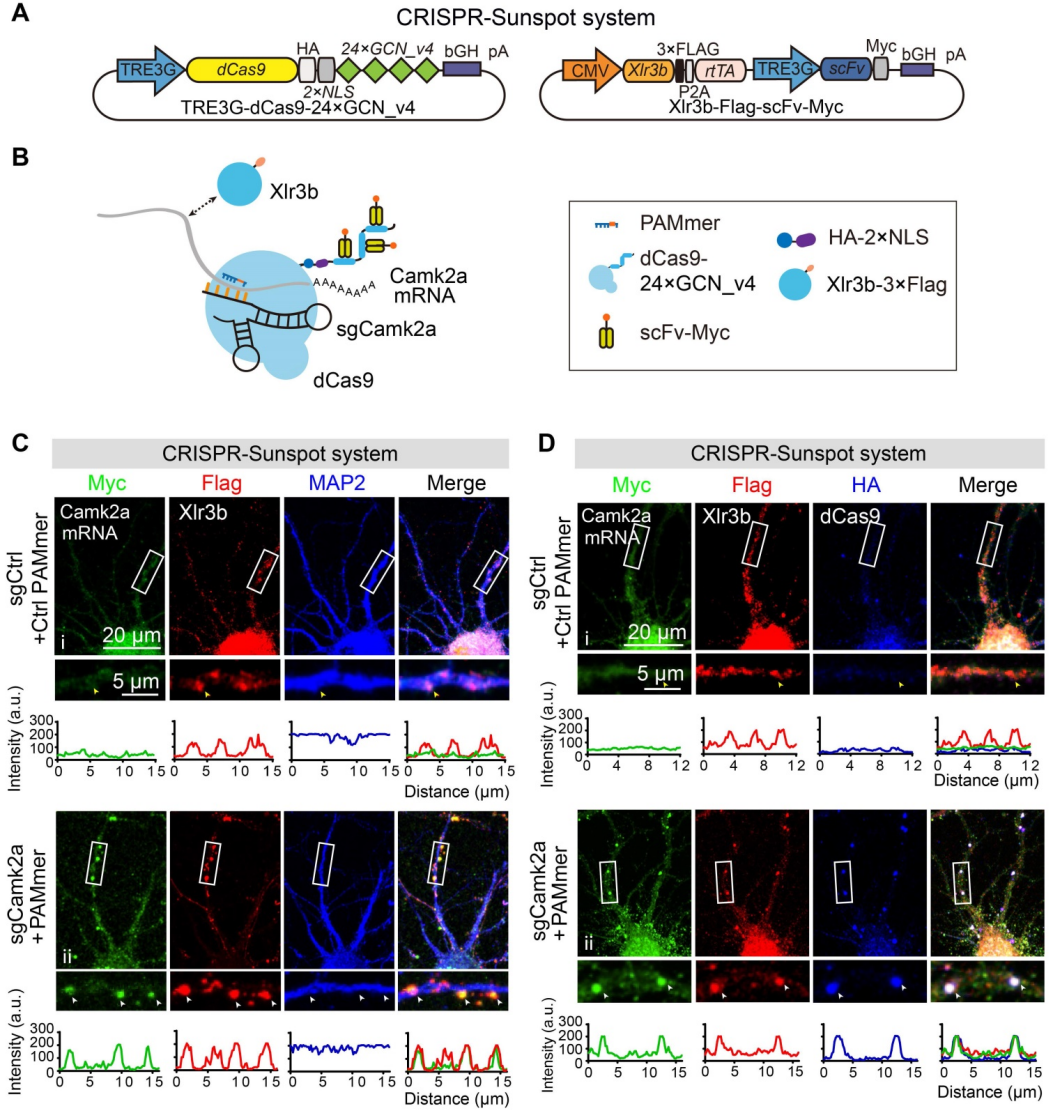
** The co-localization of endogenous *Camk2a* mRNA and Xlr3b protein in neurons revealed by CRISPR-Sunspot. (A)** Vector maps for CRISPR-Sunspot targeting *Camk2a* mRNA and Xlr3b protein overexpressing in primary neurons. Xlr3b proteins were overexpressed using the Xlr3b-Flag-scFv-Myc vector, which contains an Xlr3b expression cassette.** (B)** Schematic plots showing the components required for *Camk2a* mRNA and Xlr3b protein imaging in primary cultured neurons.** (C)** Confocal images showing co-localization of *Camk2a* mRNA (indicated by Myc) with Xlr3b protein (indicated by Flag) and MAP2 (a neuron marker) in neurons at day 8 *in vitro*. The high-magnification images in the bottom panels are enlarged from the corresponding boxed areas. Flag-positive and Myc-negative puncta in dendrites are indicated with yellow arrowheads, and Flag and Myc double-positive puncta in dendrites are indicated with white arrowheads. Plots of the arbitrary units (a.u.) along the dendrites indicated the fluorescence intensity in the high-magnification images. Scale bars, 20 µm and 5 µm (lower panel).** (D)** Confocal images showing co-localization of *Camk2a* mRNA (indicated by Myc) with Xlr3b protein (indicated by Flag) and dCas9 protein (indicated by HA) in neurons at day 8 *in vitro*. The images in the bottom panels are enlarged from the corresponding boxed areas. Flag-positive and both Myc- and HA-negative puncta in dendrites are indicated with yellow arrowheads, while Flag, Myc and HA triple-positive puncta in dendrites are indicated with white arrowheads. Plots of the arbitrary units (a.u.) along the dendrites indicated the fluorescence intensity in the high-magnification images. Scale bars, 20 µm and 5 µm (lower panel).

Of note, previous works had reported that Alpha-thalassemia X-linked intellectual disability (ATR-X) syndrome is caused by interference of overexpressed Xlr3b proteins with the transport of dendritic *Camk2a* mRNA, which leads to decreased synaptic plasticity in neurons 38. To visualize whether overexpressed Xlr3b protein was closely associated with *Camk2a* mRNA, we overexpressed Xlr3b proteins using a vector containing CMV-Xlr3b-Flag-P2A-rtTA and TRE3G-scFv-Myc cassettes (Figure 7A-B). In control sgRNA group, the green fluorescent signal of scFv-Myc was mostly restricted to nucleus, while Xlr3b was localized both in nucleus and MAP2-positive dendrites (Figure 7C, i). Notably, upon transfection with sgCamk2a and PAMmers, green fluorescent puncta were observed in the dendrites and colocalized with the red fluorescent signal of Xlr3b, indicating co-localization of *Camk2a* mRNA with Xlr3b-positive granules in the dendrites (Figure 7C, ii). Furthermore, we also directly confirmed the co-localization of *Camk2a* mRNA, Xlr3b, and dCas9 in dendrites (Figure 7D). Taken together, these results suggest that CRISPR-Sunspot can form a five-element complex containing the transported mRNA.

In summary, we have developed a CRISPR-Sunspot method to track the potential co-localization of endogenous target mRNAs with regulating proteins in neurons. This method will enable further study of pathophysiological mechanisms associated with the distribution of mRNAs and investigation into the localization between target mRNAs and regulatory proteins in a wide range of pathophysiological processes.

### CRISPR-Sunspot in combination with SunTag activation system enables imaging of activated endogenous RNA transcripts

**Figure 8:**
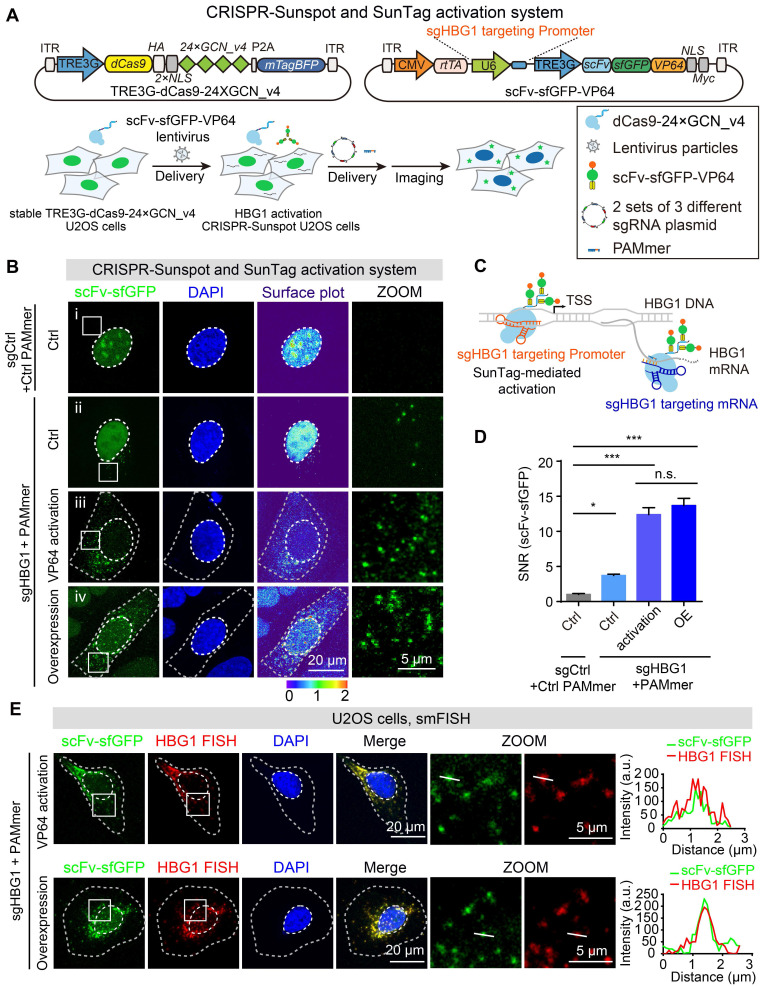
** CRISPR-Sunspot in combination with SunTag activation system for gene activation and mRNA imaging. (A)** Lentiviral vector maps for *HBG1* activation and mRNA imaging. Workflow of CRISPR-Sunspot system for imaging activated *HBG1* mRNA. Stable TRE3G-dCas9-24 × GCN_v4 U2OS cells were infected with scFv-sfGFP-VP64 lentivirus. The cells were then transfected with 2 sets of 3 different sgRNA plasmid with PAMmers targeting HBG1 mRNA for subsequent imaging.** (B)** Representative images of *HBG1* mRNA labeling using the CRISPR-Sunspot system. Scale bars, 20 µm and 5 µm (right panel). The first group (i) showed the representative images of non-activation cell lines (# Ctrl) transfected with the non-targeting control guide RNA with PAMmer. The second group (ii) showed the representative images of non-activation cell lines (# Ctrl) transfected with the sgRNA and PAMmer targeting *HBG1* mRNA. The third group (iii) showed the representative images of *HBG1* activation cell lines (# 18) transfected with sgRNA and PAMmer targeting *HBG1* mRNA. The fourth group (iv) showed the representative images of non-activation cell lines (# Ctrl) transfected by *HBG1* overexpression vector and sgRNA with PAMmer targeting *HBG1* mRNA.** (C)** Schematic plots showing the CRISPR-Sunspot system and SunTag-mediated gene activation strategies. **(D)** SNR of CRISPR-Sunspot labeling of *HBG1* mRNA in (B) (n =60, 84, 74, 60 cells).** (E)** smFISH confirms *HBG1* mRNA signals labeling by the CRISPR-Sunspot system in *HBG1* activation (by VP64) and *HBG1* overexpression (HBG1 OE) U2OS cells. Plots of the arbitrary units (a.u.) along the line indicated the fluorescence intensity in the high-magnification images. Scale bars, 20 µm and 5 µm (right panel). The data are displayed as the mean ± S.E.M. One-way ANOVA with Dunnett's multiple comparisons test was used. *P < 0.05, ***P < 0.001, n.s. not significant.

Reprogramming of CRISPR systems through the recruitment of artificial activators or repressors can be used to achieve gene activation (CRISPRa) or gene interference (CRISPRi), respectively 39,40. Traditionally, real-time fluorescent quantitative PCR (RT-qPCR) has been used to detect gene activation 41, but visualization is not available with this method. To broaden the utility of CRISPR-Sunspot, we sought to establish methods that selectively activated genes and illuminated the produced mRNA molecules, simultaneously. We chose another gene that is rarely expressed in U2OS cells, *HBG1*, as a target. Stable TRE3G-dCas9-24 × GCN_v4 cells were infected with scFv-sfGFP-VP64 lentivirus for mRNA activation (Figure 8A). VP64 is a widely used artificial transcriptional activator 39. In this procedure, we used the dCas9-24 × GCN_v4 and scFv-sfGFP-VP64 protein in combination with different sgRNAs to activate the *HBG1* transcription and image the *HBG1* transcripts, respectively. The sgHBG1 targeting promoter could specifically target the ~60 bp upstream of the transcription start site (TSS) of *HBG1*. Then, scFv-sfGFP-VP64 fusion proteins were recruited by dCas9-24 × GCN_v4 to the upstream of TSS in the *HBG1* promoter under the guidance of sgHBG1 targeting promoter for transcriptional activation. Moreover, the sgHBG1 targeting mRNA, which was in the 2 sets of 3 different sgRNA plasmids, was used for imaging (Figure 8A and 8C).

Next, to test whether CRISPR-Sunspot could elevate the mRNA levels of *HBG1*, we examined the production of *HBG1* mRNA molecules using RT-qPCR. To prevent heterogeneity among cell populations from causing variations in mRNA activation 42, we selected 40 stable cell clones for examination and found that these cells exhibited 2- to 200-fold higher mRNA levels of *HBG1* compared to control cells (infected with control lentivirus) (Figure S5A-B). In addition, we observed a positive correlation between *HBG1* activation and *VP64* expression, highlighting the importance of activator abundance for efficient mRNA activation (Figure S5A). The elevations in *HBG1* mRNA levels were also confirmed by gel electrophoresis after PCR (Figure S5C).

After achieving activation of the *HBG1* mRNA, we next evaluated whether the CRISPR-Sunspot system could recognize the produced mRNA molecules with a high SNR. When sgHBG1 were cotransfected with PAMmers, the fluorescence signal in the cytoplasm of control cells without HBG1 activation (cell lines # Ctrl, see Figure S5B) was extremely weak, presumably due to the rare expression of HBG1 (Figure 8B, ii, and 8D). Notably, upon VP64 activation and cotransfection of *HBG1* mRNA-targeting sgRNA plasmid with PAMmer, abundant fluorescent puncta appeared in the cytoplasm of cells (cell lines # 18, see Figure S5B; Figure 8B, iii, and 8D), indicating that CRISPR-Sunspot could amplify the signals of activated single-molecule mRNAs. Moreover, the significant elevation of fluorescent puncta in the cytoplasm was observed following *HBG1* overexpression (Figure 8B, iv, and D), suggesting that CRISPR-Sunspot could sensitively detect the abundance of mRNAs. Thus, we demonstrate that the CRISPR-Sunspot system can be engineered to achieve gene activation and visualize mRNAs in cells, simultaneously.

Here, smFISH were also used to validate the specificity of our CRISPR-Sunspot RNA imaging system. The distribution patterns of scFv-sfGFP targeting *HBG1* mRNA in* HBG1* activation and overexpression cells both correlate well with smFISH labeling of *HBG1* mRNA in fixed cells (Figure 8E). Furthermore, co-localization analysis revealed by Pearson's correlation (Figure S5D) indicated high specificity of the CRISPR-Sunspot system for mRNA imaging. To exclude the possibility that CRISPR-Sunspot could alter the target mRNA abundance or amount of translated protein in* HBG1* activation cell lines, we performed RT-qPCR and observed no significant differences in mRNA levels among CRISPR-Sunspot targeting *HBG1* mRNA in *HBG1* activation U2OS cells compared to the sgControl groups (Figure S5E). Consistently, western blot analysis showed that CRISPR-Sunspot did not alter the amount of translated protein (Figure S5F).

**Figure 9:**
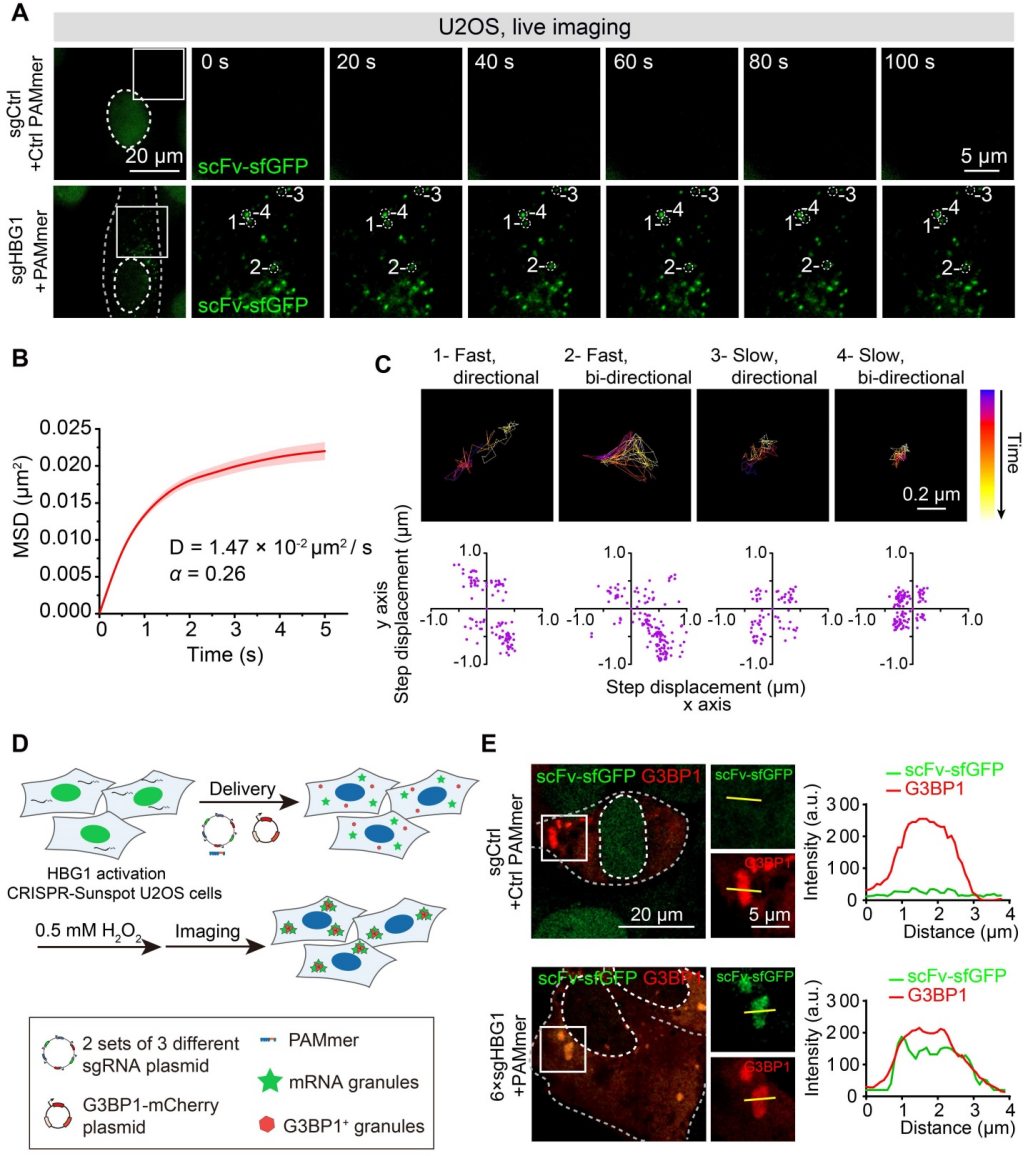
** Tracking mRNA movement and trafficking to stress granules in live cells with CRISPR-Sunspot. (A)** CRISPR-Sunspot mediated time-lapse imaging of *HBG1* mRNA in live *HBG1* activation U2OS cells. See also Video S3 and S4. Scale bars, 20 µm and 5 µm.** (B)** MSD curves and the Diffusion coefficients of single particles in the cytoplasm analyzed from at least 40 cells.** (C)** Representative trajectories of four single-particles with different movement modes. Scale bars, 0.2 µm. Scatterplots of the step displacement (δx, δy) of CRISPR-Sunspot labeling of mRNA in 120 s. Puncta were tracked every 1 s. **(D)** Schematic plots of CRISPR-Sunspot mediated imaging of *HBG1* mRNA trafficking to stress granules. **(E)** Representative images of CRISPR-Sunspot labeling of *HBG1* mRNA (green) and stress granules (G3BP1, red) upon hydrogen peroxide (0.5 mM, for 1h) treatments. Scale bars, 20 µm and 5 µm (right panel). Plots of the arbitrary units (a.u.) along the line indicated the fluorescence intensity in the high-magnification images.

Next, we also performed time-lapse tracking to study the dynamics of CRISPR-Sunspot labeling of *HBG1* mRNA in live *HBG1* activation U2OS cells (Figure 9A, Video S3 and S4). We calculated the movements of *HBG1* mRNA in U2OS cells visualized by CRISPR-Sunspot. Single-particle tracking analysis revealed that the detected scFv-sfGFP spots exhibited diffusive motion moving with diffusion coefficients (D) of 0.0147± 0.0025 μm^2^/s (Figure 9B), which is consistent with the previous study that the contents of the cytoplasm are less viscous than the nucleus 43,44. In addition, trajectories analysis revealed the confined diffusion of mRNA, which moved directionally or bi-directionally at different speeds (Figure 9C). Thus, CRISPR-Sunspot can detect dynamic changes of mRNA with high temporal resolution in living cells. We also simultaneously tracked *HBG1* mRNA using CRISPR-Sunspot and stress granules indicated by G3BP1-mCherry 45,46. We observed that the treatment of hydrogen peroxide (0.5 mM, for 1 h) to induce oxidative stress resulted in accumulation of mRNAs (indicated by scFv-sfGFP) into G3BP1-positive granules compared to non-targeting controls (Figure 9D-E).

## Discussion

Visualization of spatiotemporal distributions of transcriptional elements helps to systematically understand the biological laws between mRNA localization and its pathophysiological function 2,32. The application of the CRISPR system not only provides a variety of gene editing tools 47,48, but also offers the possibility of an in-depth exploration of RNA targeting and imaging 11-16. The previous RCas9 system has shown powerful features for tracking the high abundance transcripts 16. Here, we have for the first time reported a flexible and efficient CRISPR-Sunspot system based on the CRISPR/Cas9 and SunTag in combination with an inducible Tet-On strategy, which can successfully achieve imaging specific endogenous low-abundance RNA transcripts by using the tandem sgRNAs expression cassettes. It is noteworthy that the CRISPR-Sunspot can be used to study the location of endogenous mRNAs and the co-localization between mRNAs and proteins in polarized cells such as neurons.

For CRISPR mediated RNA imaging, several factors, including the binding affinity with targets and the expression of dCas9 and sgRNA, were initially investigated in a basic CRISPR-mediated dCas9-EGFP imaging system. dCas9 binds with a high affinity to single-stranded RNA targets matching the guide RNA sequence when the PAM is presented as a separate DNA oligonucleotide 15. In addition, dCas9 in combination with RNA pull-down also enables the isolation of a specific endogenous mRNA from cells 15. Importantly, for the binding efficiency of Suntag, fluorescence redistribution after photobleaching (FRAP) revealed that the scFv-sfGFP dissociates with a slow off-rate from the GCN4_v4 peptide array, suggesting that the scFv-sfGFP robustly bound to the GCN4_v4 peptide 19. These studies have proved that the components used in our imaging system have high-affinity binding efficiency. Here, we show that dCas9-EGFP aggregated into the nucleolus when it was redundant versus the amount of sgRNA. However, such a nucleolar signal was significantly reduced by using tandem sgRNA expression cassettes for efficient sgRNA transcription, which could induce more stable dCas9/sgRNA complexes in targeting mRNA. Our results and previous observation both clarify a critical fact that the transcriptional amount and stability of sgRNA is the crucial limiting factor for nucleic acid imaging 5,7,26. Therefore, efficient sgRNA delivery and expression or optimization of the sgRNA structure is essential aspects that are worth considering in imaging and editing. Furthermore, we control the expression of components for CRISPR imaging by combining the dCas9-fluorescent proteins with the Tet-on-induced expression system, and the results showed that inducible dCas9 system manipulating the levels of fluorescent proteins greatly enhances the efficiency of RNA imaging.

dCas9 pair with differently structured sgRNAs were designed to recruit distinct fluorescently labeled coat proteins, such as MCP-mCherry and PP7-EGFP, which was used for DNA dual-color imaging of satellite sequences 29,30. Therefore, this strategy should allow simultaneously imaging two or more kinds of mRNA species in a cell to detect transcriptome-related molecular events. Intriguingly, in our results, the *ACTB* or *GAPDH* transcripts could be labeled by dCas9 and can also be detected by the fluorescent protein recruited to the modified sgRNA in U2OS cells, simultaneously. Our method set the foundation for studying the interaction between different RNAs, such as long non-coding RNA regulating the translation or methylation of mRNA 49,50. The use of CRISPR-Sunspot in conjunction with the emerging Cas13 system 11-13 may help visualize multiple non-coding RNA or spliced mRNA in the future. Moreover, the combination of CRISPR-Sunspot and light-up RNA aptamer to achieve real-time detection of miRNA or mRNA will be another interesting research topic 51-54.

Importantly, CRISPR-Sunspot can be used to significantly amplify signals from single-molecule mRNAs with a high SNR in live cells. Until recently, several strategies such as SunTag system have been developed to enable robust fluorescent signal amplification of nucleic acid in cells 19. Moreover, SunTag system has proved to be a powerful tool to visualize the local translation by using reporters, including a series of SunTag epitopes 55. Here, CRISPR-Sunspot in combination with SunTag system successfully solves the issue of lack of tools to image low endogenous transcripts in cells which can expand dCas9-mediated imaging to explore the spatial and temporal distributions of such RNAs under physiological conditions. Recent studies showed that MoonTag was developed for visualization of translational heterogeneity 55. In the future, it is expected that the combination of CRISPR-Sunspot and MoonTag technology will enable simultaneous visualization of translated mRNA and protein or alternatively spliced mRNA.

Interestingly, in CRISPR-Sunspot, SunTag system can be used to enhance gene transcription and improve imaging sensitivity for low-abundance endogenous transcripts, simultaneously. In our experiments, gene activation was achieved through CRISPRa, in which scFv-sfGFP-VP64 was recruited to the gene promoter. Further, transcriptional output could be simultaneously detected through robust fluorescent signal amplification via sfGFP. Therefore, our imaging system expects to not only provide a visualized reference for the selection of activation sites on the promoter of the candidate gene, but also be used for the RNA transcriptional kinetics studies in the future.

CRISPR-Sunspot also provides a powerful tool to study the localization of endogenous mRNA in neurons. In the polarized cells, especially neurons, some mRNA molecules such as *ACTB*, *Camk2a,* and *Arc* travel along dendrites from the nucleus to subcellular destinations, where they will be subject to the local protein translation and execute functions for synapse formation 35. Interestingly, most of the neuronal mRNA species in the hippocampus are localized and enriched in axons or dendrites compared with the soma 32. Abnormal aggregation of mRNA with microsatellite repeat expansions is also related to many neurological diseases, such as C9orf72-linker amyotrophic lateral sclerosis (C9-ALS) 34. The defective transportation of these transcripts has also been found associated with neurodegenerative diseases including ATR-X syndrome 38. In previous work, Shioda et al. imaged MS2-GFP-labeled *Camk2a* mRNA and mCherry labeling of Xlr3b to determine whether Xlr3b protein could co-transported with *Camk2a* mRNA into dendrites 38. Traditional RNA imaging methods require inserting specific multiplexes MS2 sequences at the 3' end of the coding gene, which was widely used for various tracking of mRNA. These RNA imaging methods require modification of genomic DNA or the introduction of an exogenous sequence to the RNA of interest, and overexpression of the target RNA carrying MS2 elements by vectors 56,57. However, based on our results, CRISPR-Sunspot system provides a powerful tool to study the localization of endogenous mRNA in different cell types by the specific transcripts recognition.

## Conclusions

Fluorescence *in situ* hybridization is widely used to study the subcellular distributions of various mRNA species with implications for cellular development, neural activity, or pathological processes; however, this method is limited to fixed cells and complicated operations. The mRNA imaging in live cells is an essential approach for studying the features of mRNAs at different spatial and temporal scales. Here, CRISPR-Sunspot was developed as a flexible method of mRNA targeting that allows efficient labeling of endogenous transcripts present at low abundances. In addition, we have demonstrated the utility of CRISPR-Sunspot for the labeling of endogenous mRNAs in neurons. Moreover, the use of CRISPR-Sunspot in combination with CRISPR activation provides a foundation for research on the relationship of transcription and distributions of mRNAs in cells. Our method can potentially support the imaging of various mRNA species as well as pathological investigation of diseases caused by aberrant mRNA molecules.

## Supplementary Material

Supplementary figures and tables.

Supplementary video S1.

Supplementary video S2.

Supplementary video S3.

Supplementary video S4.

## Abbreviations

PB: PiggyBac
smFISH: single molecule fluorescence *in situ* hybridization
CRISPR: clustered regularly interspaced short palindromic repeats
dCas9: nuclease-deactivated Cas9
sgRNA: single guide RNA
SNR: signal-to-noise ratio
CRISPR-Sunspot: Suntag-mediated single molecule RNA snapshot
E18: embryonic day 18
BSA: bovine serum albumin
s.e.m.: standard error of the mean
NLS: nuclear localization signal
CRISPRa: CRISPR activation
CRISPRi: CRISPR interference
RT-qPCR: real-time fluorescent quantitative PCR
TDP-43: trans-active response DNA-binding protein 43
Camk2a: Ca^2+^/calmodulin-dependent protein kinase 2a
ATR-X: alpha-thalassemia X-linked intellectual disability
rtTA: reverse tetracycline-controlled transactivator
C9-ALS: C9orf72-linker amyotrophic lateral sclerosis
