# Supplementary material for: CRISPR-Sunspot: Imaging of endogenous low-abundance RNA at the single-molecule level in live cells
Source: Theranostics. 2020 Sep 2;10(24):10993–1012. doi: 10.7150/thno.43094 (PMC7532675; doi:10.7150/thno.43094)
Supplement: Supplementary file 1 — Supplementary figures and tables. [file thnov10p10993s1.pdf]

Supplementary Information

CRISPR-Sunspot: Imaging of endogenous low-abundance RNA at the single-molecule level in live cells

Ning-He Sun[1,2,3,¶], Dan-Yang Chen[1,2,3,¶], Lu-Peng Ye[4], Gang Sheng[2], Jun-Jie Gong[2], Bao-Hui Chen[5], Ying-Mei Lu[1,*], Feng Han[2,6,*]

1. Department of Physiology, School of Basic Medical Sciences, Nanjing Medical University, Nanjing, 211166, China

2. Key Laboratory of Cardiovascular & Cerebrovascular Medicine, Drug Target and Drug Discovery Center, School of Pharmacy, Nanjing Medical University, Nanjing, 211166, China

3. Institute of Pharmacology and Toxicology, College of Pharmaceutical Sciences, Zhejiang University, Hangzhou, 310058, China

4. Department of Genetics, Yale University School of Medicine, New Haven, CT, USA.

5. Department of Cell Biology, School of Medicine, Zhejiang University, Hangzhou, 310058, China

6. Center for Global Health of Nanjing Medical University, Nanjing, 211166, China.

Supplementary Figures and Figure Legends

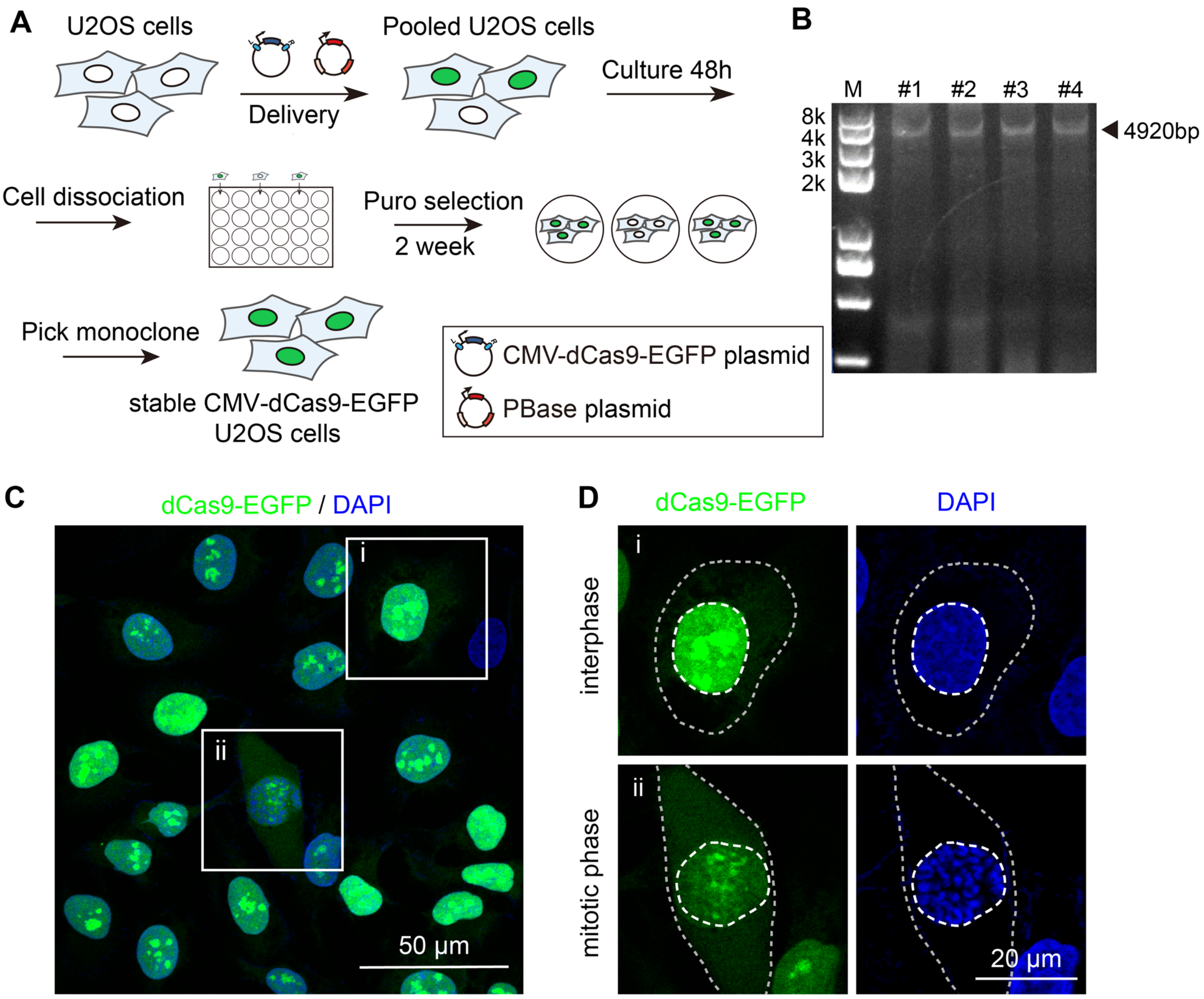

Figure S1. Construction of stable dCas9-EGFP expressing cells for RNA imaging. (A) Workflow for screening of stable CMV-dCas9-EGFP U2OS monoclonal cells. The U2OS cells were transfected with CMV-dCas9-EGFP and PBase vectors, followed by puromycin selection. Then optimal clonal cells could be transfected with vectors carrying sgRNAs and PAMmer for subsequent imaging. **(B)** Genomic DNA from representative clonal cells was purified and used as the PCR template. Representative gels of PCR products are shown to indicate the correct insertions of dCas9-EGFP coding gene. The size of all PCR products was

correct as expected. **(C)** Representative images of dCas9-EGFP expression driven by CMV. Scale bars, 50 μm. **(D)** Representative higher magnification of boxed images of dCas9-EGFP expression at different stages of mitosis in C: interphase **(i)**, mitotic phase **(ii)**. Scale bars, 20 μm.

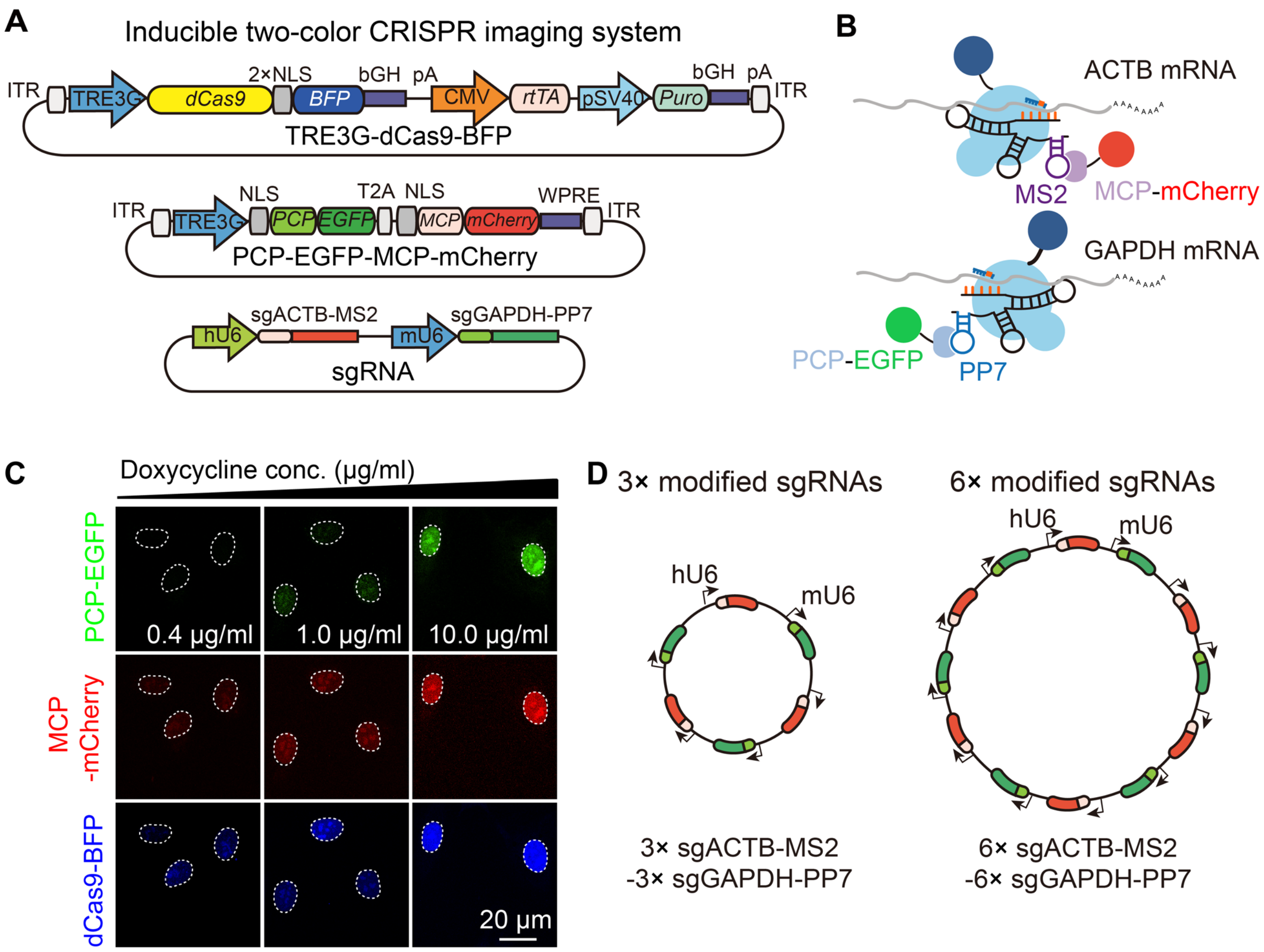

Figure S2. Construction of stable Inducible two-color CRISPR imaging system for mRNA labeling. **(A)** Diagram of the expression vectors for the Inducible two-color CRISPR imaging system. The PCP and MCP fused to EGFP or mCherry, respectively, were expressed under the TRE3G promoter. The sgRNA scaffolds contained PP7 or MS2 stem-loops, respectively. **(B)** Schematics showing the components required for *ACTB* and *GAPDH* mRNA imaging. dCas9 allows binding of sgRNAs to the target mRNAs, and each sgRNAs containing PP7 or MS2 stem-loops recruit PCP-EGFP or MCP-mCherry, respectively. **(C)** dCas9-BFP (blue), MCP-mCherry (red), and PCP-EGFP (green) were expressed under the TRE3G promoter in the presence of different doxycycline concentrations. Scale bars, 20 μm.

(D) 3 or 6 × modified sgRNAs targeting *ACTB* or *GAPDH* mRNA were constitutively transcribed from the human or mouse U6 promoter, respectively.

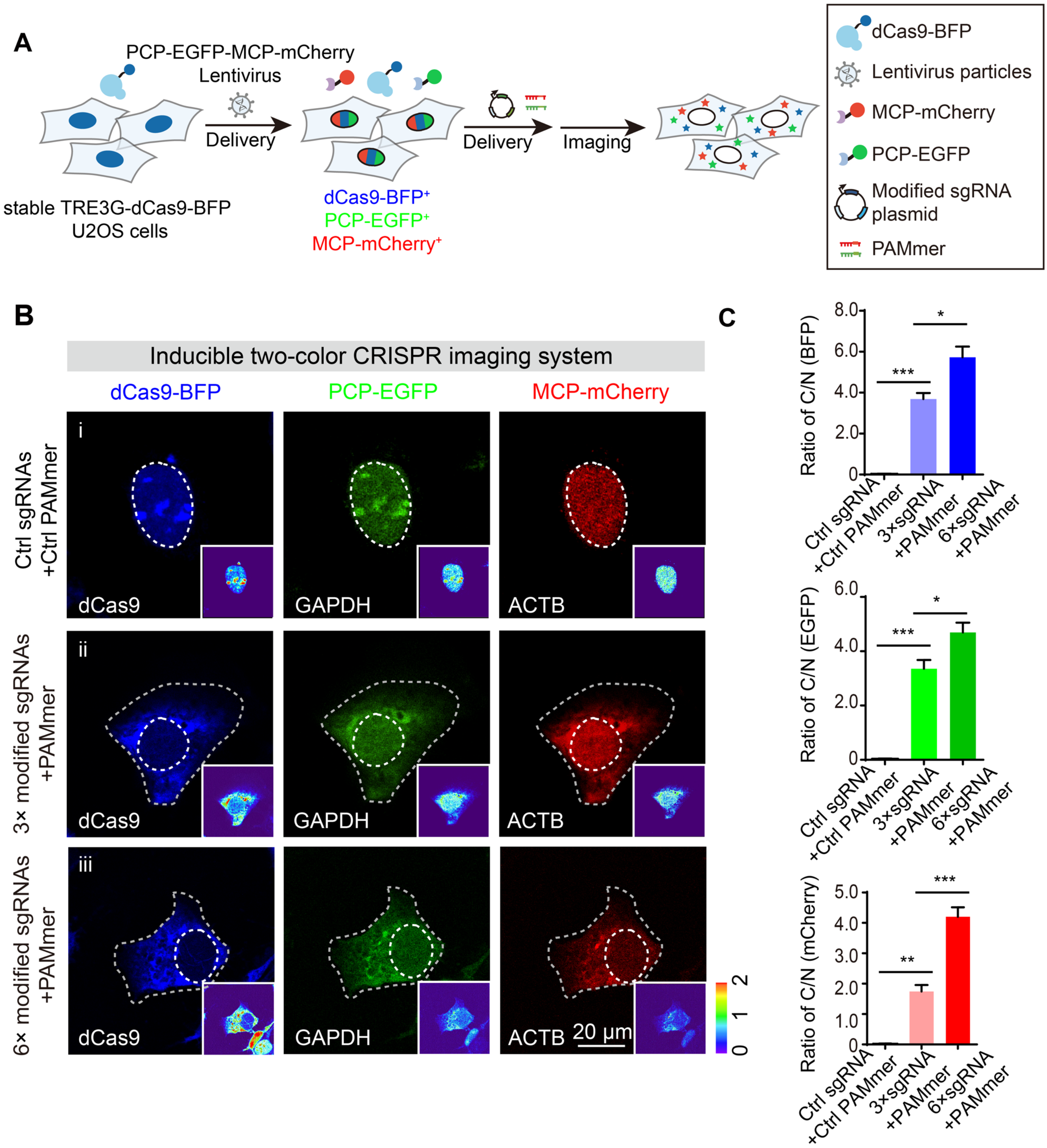

Figure S3. Two-color labeling of mRNAs by the Inducible two-color CRISPR imaging system. (A) Workflow for two-color mRNA imaging. Stable dCas9-BFP U2OS cells were infected with PCP-EGFP-MCP-mCherry lentivirus. dCas9-BFP, PCP-EGFP, and MCP-mCherry triple-positive U2OS clonal cells were selected. The cells were then transfected with sgRNA and PAMmers for subsequent mRNA imaging. **(B)** Representative

images of *GAPDH* and *ACTB* mRNA labeling in stable U2OS cells transfected with modified sgRNAs and PAMmers. The localization of dCas9-BFP (blue), PCP-EGFP (green), and MCP-mCherry (red) in U2OS cells are shown. Scale bars, 20 μm. **(C)** The fluorescence intensity of two-color mRNA imaging was quantified by measuring the ratio of C/N of BFP, EGFP, and mCherry (n = 83, 75, 79 cells). The data are displayed as the mean ± S.E.M. One-way ANOVA with Dunnett's multiple comparisons test was used. $^{*}P < 0.05$, $^{**}P < 0.01$, $^{***}P < 0.001$.

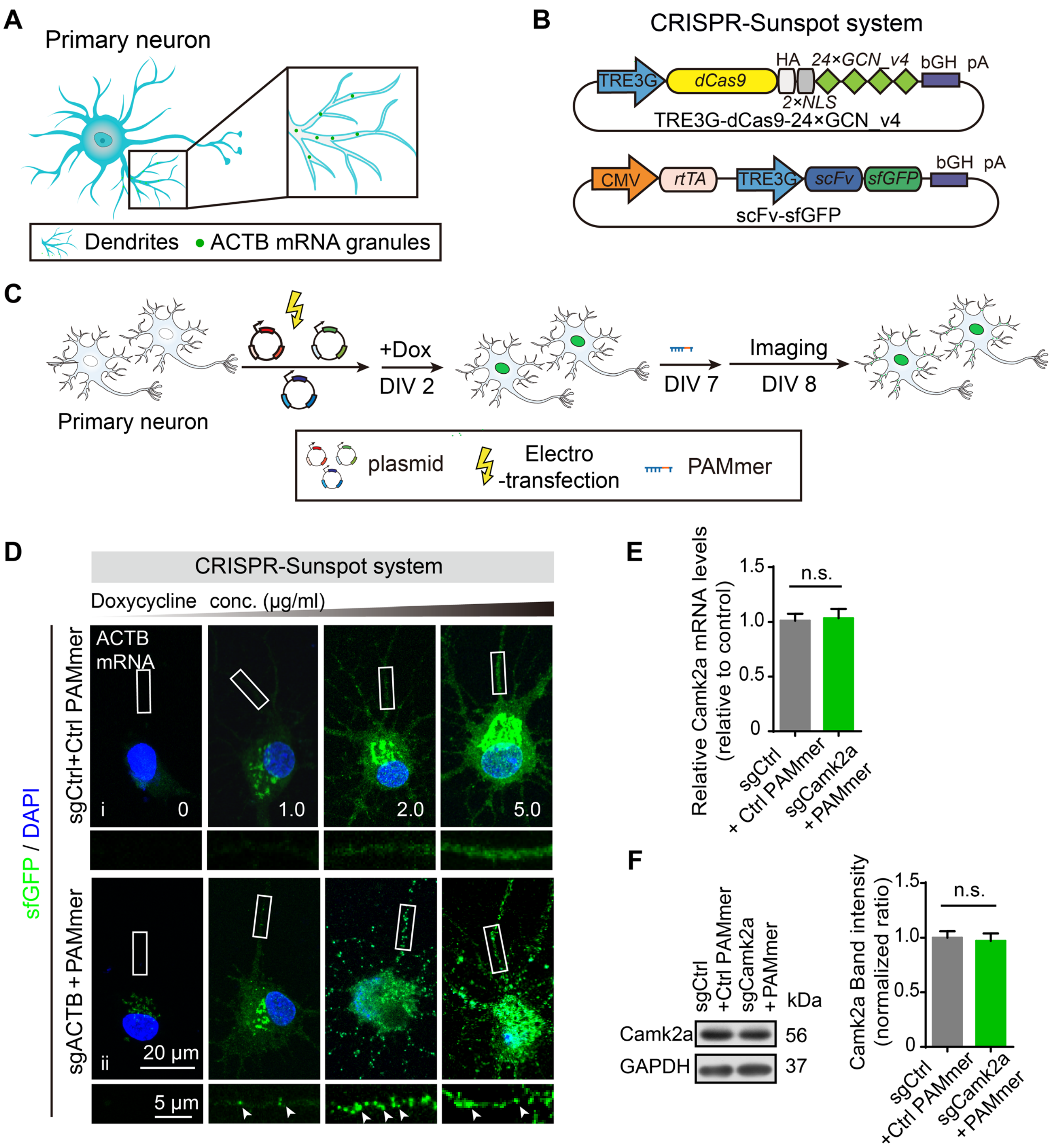

Figure S4. Application of the CRISPR-Sunspot system to image *ACTB* mRNA in dendrites of neurons. (A) Schematics showing the localization of *ACTB* mRNA granules in dendrites of neurons. **(B)** Diagram of the expression vectors for the CRISPR-Sunspot system in primary neurons. **(C)** Workflow for the CRISPR-Sunspot system in primary neurons. The cells were transfected with imaging vectors expressing dCas9-24 × GCN_v4, scFv-sfGFP, and

sgRNA using a Nucleofector device (Lonza). At day *in vitro* (DIV) 2, the neurons were treated with doxycycline. At DIV 7, they were transfected with PAMmers. At DIV 8, the neurons were used for imaging. **(D)** Cultured hippocampal neurons were transfected with control sgRNA (upper panel) or sgCamk2a (lower panel) with imaging plasmids and PAMmers then treated with different concentrations of doxycycline. The images in the bottom panels are enlarged from the corresponding boxed areas. The arrowheads indicate GFP-positive puncta. Scale bars, 20 μm (upper panel) and 5 μm (lower panel). **(E)** RT-qPCR analysis of the level of *Camk2a* mRNA in cultured neurons transfected with control sgRNA or sgCamk2a with PAMmers. sgCtrl: n = 4 groups; sgCamk2a: n = 4 groups. **(F)** Immunoblot analysis of Camk2a protein level in cultured neurons transfected with control sgRNA or sgCamk2a with PAMmers. Quantitative analysis of the results and presented in the bar graph as the densitometry ratio of the control from four independent experiments. The data are displayed as the mean ± S.E.M. An unpaired t test was used. n.s. not significant.

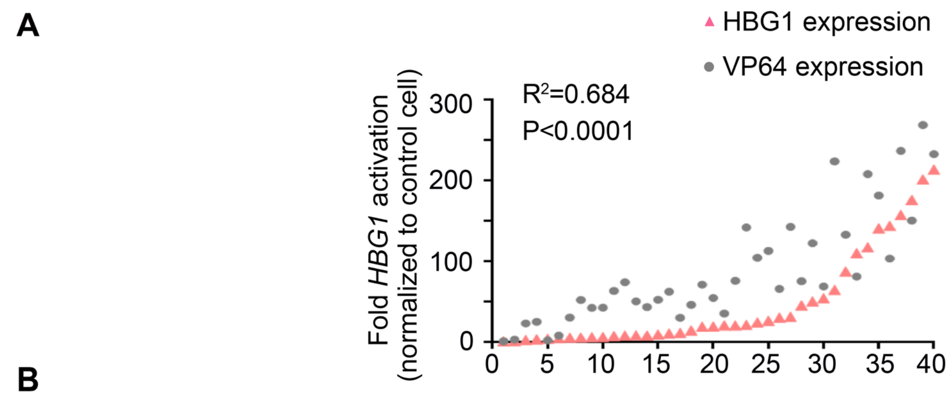

B

X-axis value	Serial number of cell line	Fold change	X-axis value	Serial number of cell line	Fold change	X-axis value	Serial number of cell line	Fold change	X-axis value	Serial number of cell line	Fold change
1	# Ctrl	1.00	11	# 20	7.37	21	# 23	20.58	31	# 39	64.52
2	# 31	1.35	12	# 15	8.06	22	# 10	20.56	32	# 19	87.34
3	# 14	2.69	13	# 8	8.35	23	# 5	21.36	33	# 6	110.23
4	# 24	3.43	14	# 11	8.21	24	# 9	24.35	34	# 33	117.25
5	# 35	4.15	15	# 38	9.25	25	# 32	26.02	35	# 12	140.58
6	# 13	4.93	16	# 30	10.57	26	# 25	30.25	36	# 4	143.96
7	# 1	5.31	17	# 34	11.35	27	# 7	31.26	37	# 17	157.33
8	# 37	5.62	18	# 29	14.35	28	# 21	45.32	38	# 27	175.86
9	# 26	6.08	19	# 3	19.21	29	# 28	50.31	39	# 16	201.36
10	# 22	6.32	20	# 2	19.32	30	# 36	54.36	40	# 18	213.56

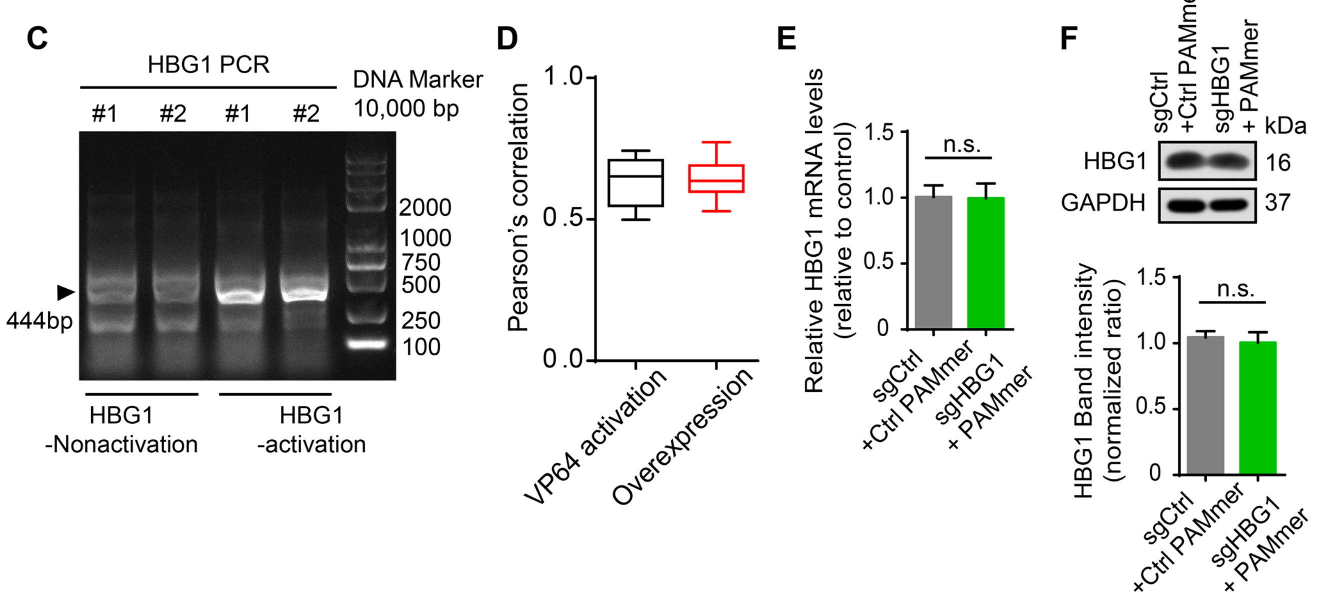

Figure S5. Elements for *HBG1* gene activation and mRNA imaging. (A) 40 stable cell

clones after lentivirus infection for an examination of HBG1 transcriptional activation compared to control cells (infected with control lentivirus). The triangles (pink) and dots (black) indicated the levels of *HBG1* or *VP64* mRNA in the stable cell line, respectively. **(B)** The exact fold change values of *HBG1* mRNA level shown in **A.** The x-axis value related to the serial number of cell lines. **(C)** The elevations in *HBG1* mRNA levels were confirmed by gel electrophoresis after PCR. Total mRNA from four monoclonal cells was purified and reverse transcribed as the PCR template. Representative gels of PCR products are shown to indicate the elevations in *HBG1* mRNA after activation. The arrowheads indicate the expected PCR products. **(D)** Co-localization analysis between scFv-sfGFP targeting *HBG1* mRNA and smFISH labeling of *HBG1* mRNA in HBG1 activation (by VP64) and HBG1 overexpression cells, quantified per cell revealed by Pearson's correlation. Box plots are represented from 39, 39 cells, respectively. **(E)** RT-qPCR analysis of *HBG1* mRNA level in *HBG1* activation CRISPR-Sunspot U2OS cells transfected with control sgRNA or sgHBG1 with PAMmers. sgCtrl: n = 4 groups; sgHBG1: n = 4 groups. **(F)** Immunoblot analysis of HBG1 protein level in HBG1 activation CRISPR-Sunspot U2OS cells transfected with control sgRNA or sgHBG1 with PAMmers. Quantitative analyses of the results and presented in the bar graph as the densitometry ratio of the control from four independent experiments. The data are displayed as the mean ± S.E.M. An unpaired t test was used. n.s. not significant.

Video S1.

Live imaging in neurons using CRISPR-Sunspot with non-targeting sgControl and control PAMmer, related to Figure 6E. Scale bar, 10 μm.

Video S2.

Live imaging of the *Camk2a* mRNA in neurons using CRISPR-Sunspot with sgCamk2a and PAMmer, related to Figure 6E. Scale bar, 10 μm.

Video S3.

Live imaging in U2OS cells using CRISPR-Sunspot with non-targeting sgControl and control PAMmer, related to Figure 9A. Scale bar, 20 μm.

Video S4.

Live imaging of the *HBG1* mRNA in HBG1 activation U2OS cells using CRISPR-Sunspot with sgHBG1 and PAMmer, related to Figure 9A. Scale bar, 20 μm.

Table S1. Imaging system used in this study.

Applications	Imaging system	Fluorescence
Figure 2D, G	dCas9-EGFP imaging system	EGFP
Figure 3E	Inducible dCas9-EGFP imaging system	EGFP
Figure 4D	Inducible dCas9-EGFP imaging system	EGFP
Figure 4E	CRISPR-Sunspot system	sfGFP
Figure 5A, D	CRISPR-Sunspot system	sfGFP
Figure 6C, i-ii, and 6D-E	CRISPR-Sunspot system	sfGFP
Figure 6C, iii-iv	Inducible dCas9-EGFP imaging system	EGFP
Figure 7C-D	CRISPR-Sunspot system	sfGFP
Figure 8B, E	CRISPR-Sunspot and SunTag activation system	sfGFP
Figure 9A, E	CRISPR-Sunspot and SunTag activation system	sfGFP
Figure S2C	Inducible two-color CRISPR imaging system	BFP\EGFP\mCherry
Figure S3B	Inducible two-color CRISPR imaging system	BFP\EGFP\mCherry
Figure S4D	CRISPR-Sunspot system	sfGFP

Table S2. sgRNA sequence used in this study.

Target	Target sequence (5'-3')	sgRNA sequence (5'-3')
ACTB	UGGAGCGAGCAUCCCCCAAA	UUUGGGGGAUGCUCGCUCCA
GAPDH	CCCUCACUGCUGGGGAGUCC	GGACUCCCCAGCAGUGAGGG
HBS1L-1	CUAAAAACUAGUAAAUAAAU	AUUUAUUUACUAGUUUUUAG
HBS1L-2	AUAACUGAUCCGUUGGGUGU	ACACCCAACGGAUCAGUUAU
HBS1L-3	AACAAGAAAAUUAAAUGAAG	CUUCAUUUAAUUUUCUUGUU
HBG1 (Promoter)	CCAGTGAGGCCAGGGGCCGG (CGG)	CCAGUGAGGCCAGGGGCCGG
HBG1-1	GCUUUAUUCUGCAAGCAAUA	UAUUGCUUGCAGAAUAAAGC
HBG1-2	CCCUGAGGUGCAGGCUUCCU	AGGAAGCCUGCACCUCAGGG
HBG1-3	CUGAGAACUUCAAGCUCCUG	CAGGAGCUUGAAGUUCUCAG
ACTB-1 (neuron)	GGCAAUGAGCGGUUCCGAUG	CAUCGGAACCGCUCAUUGCC
ACTB-2 (neuron)	AGAUUACUGCCCUGGCUCCU	AGGAGCCAGGGCAGUAAUCU
ACTB-3 (neuron)	UUACACCCUUUCUUUGACAA	UUGUCAAAGAAAGGGUGUAA
Camk2a-1 (neuron)	AGAGCAGCUGAUCGAAGCCA	UGGCUUCGAUCAGCUGCUCU
Camk2a-2 (neuron)	UGACAGCCUUUGAACCGGAG	CUCCGGUUCAAAGGCUGUCA
Camk2a-3 (neuron)	UCAGUACCUGGAUGCGGGUG	CACCCGCAUCCAGGUACUGA
Control-1	CAUGGCAUUCCACUUAUCAC	GUGAUAAGUGGAAUGCCAUG
Control-2	CACUAUCGGAAGUUCACCAG	CUGGUGAACUUCCGAUAGUG
Control-3	GAACCACCAGGCUAUAUCUG	CAGAUAUAGCCUGGUGGUUC

Table S3. PAMmer sequence used in this study.

Target	sequence (5'-3')
ACTB	mUCmGCmUCmCAmUGGmGAmCTmGCmUGmUCmACmCTmUC
GAPDH	mAGmUGmAGmGGmCGGmCTmCTmCTmUCmCTmCTmUGmUG
HBS1L-1	mGTmUTmUTmAGmUGGmUCmCCmUCmUGmAGmCTmUTmAG
HBS1L-2	mUCmAGmUTmATmUGGmUGmUCmUCmCCmCAmGTmACmAT
HBS1L-3	mUTmCTmUGmUTmUGGmUTmUCmCAmUCmUTmUTmGCmUG
HBG1-1	mAAmUAmAAmGCmCGGmUCmCTmUGmAAmAGmCTmCTmGA
HBG1-2	mCCmUCmAGmGGmUGGmAAmUTmCTmUTmGCmCGmAAmAT
HBG1-3	mGTmUCmUCmAGmUGGmCCmACmATmGCmAGmCTmUGmUC
ACTB-1 (neuron)	mUCmATmUGmCCmUGGmAGmUGmATmGAmCCmUGmACmCG
ACTB-2 (neuron)	mAGmUAmATmCTmCGGmUCmUGmCAmUCmCTmGTmCAmGC
ACTB-3 (neuron)	mGGmGTmGTmAAmCGGmGCmAGmCTmCAmGTmAAmCAmGT
Camk2a-1 (neuron)	mGCmUGmCTmCTmUGGmACmUTmUGmATmAAmUTmUCmCT
Camk2a-2 (neuron)	mGGmCTmGTmCAmUGGmCAmGGmGTmCGmCAmCAmUCmUT
Camk2a-3 (neuron)	mGGmUAmCTmGAmUGGmATmGCmGGmATmGTmAGmGCmGA
Control-1	mATmGCmCAmUGmUGGmGCmUGmUCmAAmAAmUTmGAmGC
Control-2	mCGmATmAGmUGmCGGmGTmGTmUGmAAmUGmATmUTmCC
Control-3	mGGmUGmGTmUCmUGGmCGmCGmCAmUTmUTmUAmUTmGC

Table S4. sgRNA scaffold sequence used in this study.

sgRNA scaffold	sequence (5'-3')
sgRNA $^{(F+E)}$	GUUUAAGAGCUAUGCUGGAAACAGCAUAGCAAGUUUAAAUAAGG CUAGUCCGUUAUCAACUUGAAAAAGUGGCACCGAGUCGGUGCUU UUUUU
sgRNA-MS2	GUUUUAGAGCUAGGCCAACAUGAGGAUCACCCAUGUCUGCAGGG CCUAGCAAGUUAAAAUAAGGCUAGUCCGUUAUCAACUUGGCCAA CAUGAGGAUCACCCAUGUCUGCAGGGCCAAGUGGCACCGAGUCGG UGCUUUUUUU
sgRNA-PP7	GUUUUAGAGCUAUAAGGAGUUUAUAUGGAAACCCUUAUAGCAAG UUAAAAUAAGGCUAGUCCGUUAUCAACUUGGCCUAAGGAGUUUA UAUGGAAACCCUUAGGCCAAGUGGCACCGAGUCGGUGCUUUUUU U

Table S5. Primers sets for RT-qPCR in this study.

Target	Forward primer (5' to 3')	Reverse primer (5' to 3')
HBS1L	ACATCCTCAGATCTCCCATCCAG	CCCCTACCTTAAACCCCAATCC
HBG1	AACCCCAAAGTCAAGGCACA	CATCTTCTGCCAGGAAGCCT
VP64	AGATATGCTTGGTTCAGACGCG	CAAAGTCATCTAGGGCATCGGAG
Camk2a	TCCGGAGGGAAGAGTGGAGGAAAC	GCTGTCATTCCAGGGTCGCACAT
GAPDH	TTTGGTCGTATTGGGCGCCTGG	CTCAGCCTTGACGGTGCCATGG
